# Candidate gene-environment interactions in substance abuse: A systematic review

**DOI:** 10.1371/journal.pone.0287446

**Published:** 2023-10-31

**Authors:** Zheng Jiang, Zidong Chen, Xi Chen

**Affiliations:** 1 Jockey Club School of Public Health and Primary Care, Faculty of Medicine, The Chinese University of Hong Kong, Hong Kong, Hong Kong; 2 Department of Sociology and Social Policy, Lingnan University, Tuen Mun, Hong Kong; University of Wisconsin-Green Bay, UNITED STATES

## Abstract

**Background:**

The abuse of psychogenic drugs can lead to multiple health-related problems. Genetic and environmental vulnerabilities are factors in the emergence of substance use disorders. Empirical evidence regarding the gene–environment interaction in substance use is mixed. Summaries of the latest findings from a candidate gene approach will be useful for revealing the significance of particular gene contributions. Thus, we aim to identify different gene–environment interactions in patterns of substance use and investigate whether any effects trend notably across different genders and races.

**Methods:**

We reviewed published studies, until March 1, 2022, on substance use for candidate gene–environment interaction. Basic demographics of the included studies, target genes, environmental factors, main findings, patterns of gene–environment interaction, and other relevant information were collected and summarized.

**Results:**

Among a total of 44 studies, 38 demonstrated at least one significant interaction effect. About 61.5% of studies on the *5-HTTLPR* gene, 100% on the *MAOA* gene, 42.9% on the *DRD2* gene, 50% on the *DRD4* gene, 50% on the *DAT* gene, 80% on the *CRHR1* gene, 100% on the *OPRM1* gene, 100% on the *GABRA1* gene, and 50% on the *CHRNA* gene had a significant gene–environment interaction effect. The diathesis–stress model represents a dominant interaction pattern (89.5%) in the studies with a significant interaction effect; the remaining significant effect on substance use is found in the differential susceptibility model. The social push and swing model were not reported in the included studies.

**Conclusion:**

The gene–environment interaction research on substance use behavior is methodologically multidimensional, which causes difficulty in conducting pooled analysis, or stated differently–making it hard to identify single sources of significant influence over maladaptive patterns of drug taking. In decreasing the heterogeneity and facilitating future pooled analysis, researchers must (1) replicate the existing studies with consistent study designs and measures, (2) conduct power calculations to report gene–environment correlations, (3) control for covariates, and (4) generate theory-based hypotheses with factorial based experiments when designing future studies.

## Introduction

According to the United Nations Office on Drugs and Crime, 275 million people used licit or illicit drugs in 2020 worldwide: 22.3% of them used tobacco [1], and nearly 4% of all deaths are related to alcohol misuse [2]. Meanwhile, 36 million people are suffering from substance use disorders [3]. Taking the United States as an example, 11.7% of persons aged over 12 used illicit drugs in 2018 [4]. Substance use imposes a great burden on individuals and countries as it leads to various health problems, including substance dependence, cardiovascular diseases, and and other psychological illnesses. Thus, prevention and treatment of substance use remain as a public health priority.

It is well-established that both genetic and environmental factors contribute to substance use behavior [5]. Twin studies used to investigate the heritability of substance use behavior [6] revealed that heritability only partially explains (about 30%–75%) substance use, abuse, and dependence [5], when considering the possibility that heritability scores are vulnerable to exaggeration, there remains considerable room for environmental factors, including an interaction between genetic and environmental factors. Environmental factors refer to the physical and social environment in which people live and conduct their daily activities [5, 7]. In this review, environmental factors include both individual- and societal-level variables, such as stressful life events and familial relationships. In most cases, analog scales are used to to evaluate, quantify, and operationally define the level or intensity of an environment variable.

Like substance abuse, genetic and environmental factors also contribute to psychiatric outcomes with varying effects in adulthood. For instance, although 5-HTTLPR (serotonin transporter gene-linked polymorphic region) variation was found to be significantly associated with anxiety and depression, it only accounted for 7%–9% of inherited variance in anxiety-related personality traits [7]. Social factors, such as stressful life events, may also play an important role in mental health and interact with genetic factors. A same-sex twin longitudinal study [8] revealed a causal relationship between the experience of stressful life events and the onset of major depression. In addition, there may be a significant interaction between genetic and environmental factors on the expression of psychiatric disorders. A study on depression showed that *s/s* homozygotes of *5-HTTLPR* increased depressive symptoms only when early traumatic life events occurred [9]. Similarly, people who experienced childhood maltreatment with a genotype of high levels of monoamine oxidase A enzyme (*MAOA*) expression had a weaker association with antisocial problems than people with low *MAOA* activity [10].

Exposure to negative psychosocial factors may also increase the risk of developing substance use or substance abuse behavior [11–13], although some studies provide no significant effects here, because people exposed to similar difficult environments, do not necessarily develop substance use behaviors[14]. More clarity on the interaction between environment and biology in the ontogeny of substance use disorders is therefore required, and more specificity to this question can be obtained by asking: “Are there specific factors that relate to the genetic effect or the psychosocial effect in the onset and maintenance of the problems associated with substance use? And equally important is the question of how robustly and precisely does any one factor contribute?” [15]. Prior research has revealed four major types of the gene–environment interaction (G×E) effect: the diathesis–stress model, the differential susceptibility model, the “social push” model, and the “swing” model.

**Fig 1:**
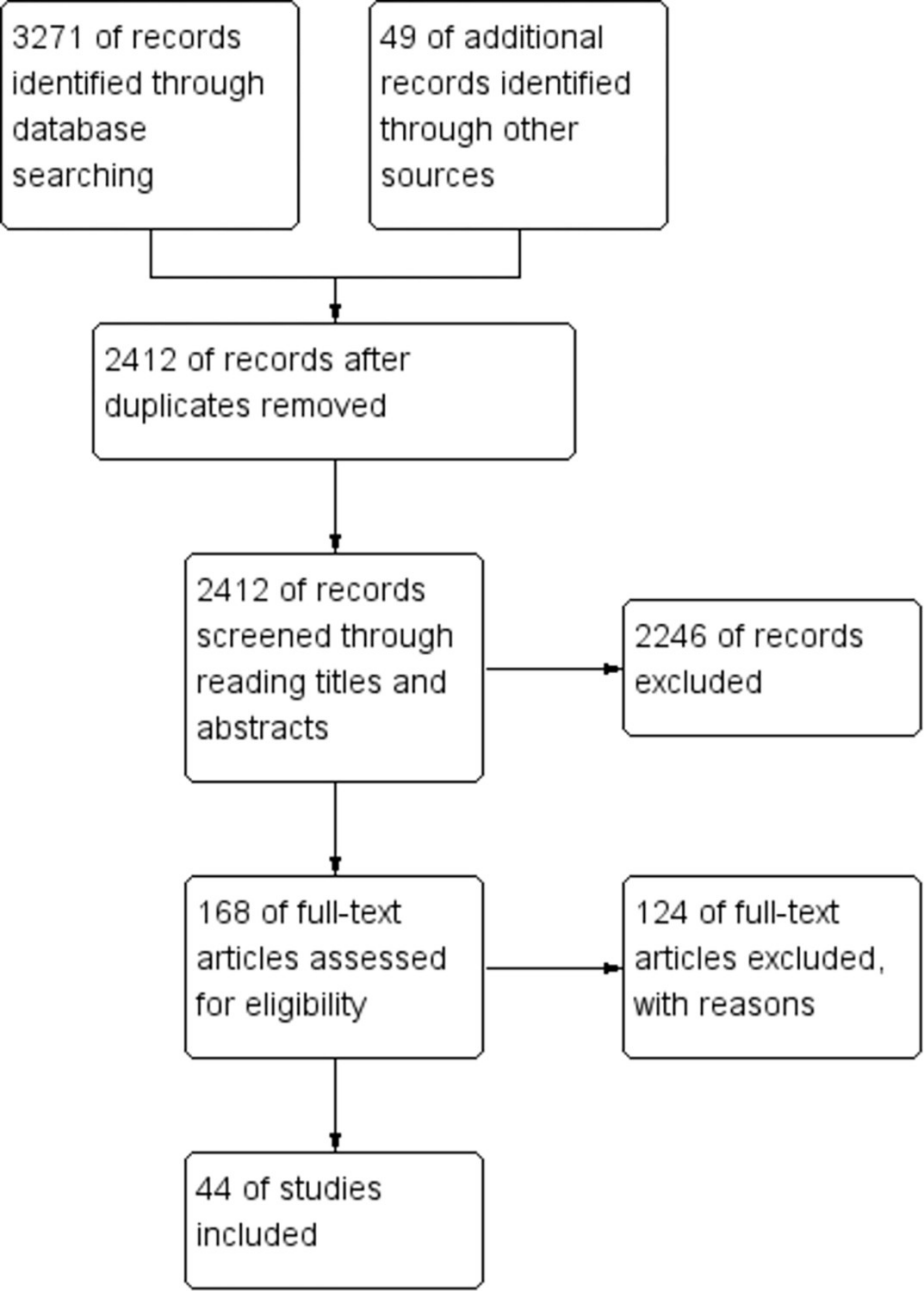
Diathesis–stress model.

**Fig 2:**
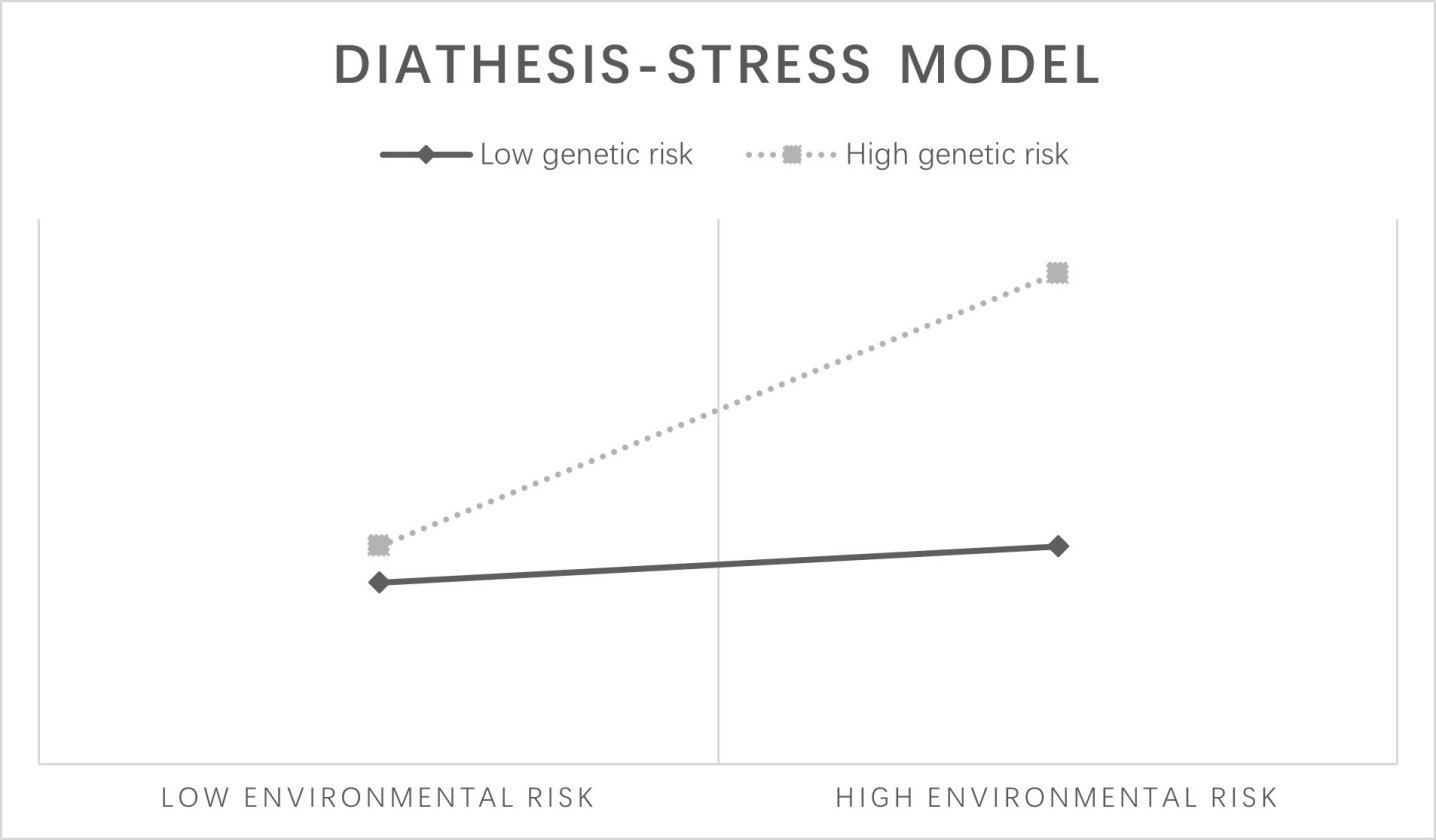
Differential susceptibility model.

The diathesis–stress model (Fig 1) [16–19] suggests that individuals with high genetic risk are more likely to experience adverse outcomes when exposed to high-risk environments. This model is commonly reported in G×E effects on patterns of substance use behavior [17–19]. The differential susceptibility model (Fig 2) [20] indicates that though individuals with a certain genetic predisposition have greater vulnerability to high-risk environments, and they can also obtain more benefits from positive environments. This model was also observed in many substance use studies [21, 22].

**Fig 3:**
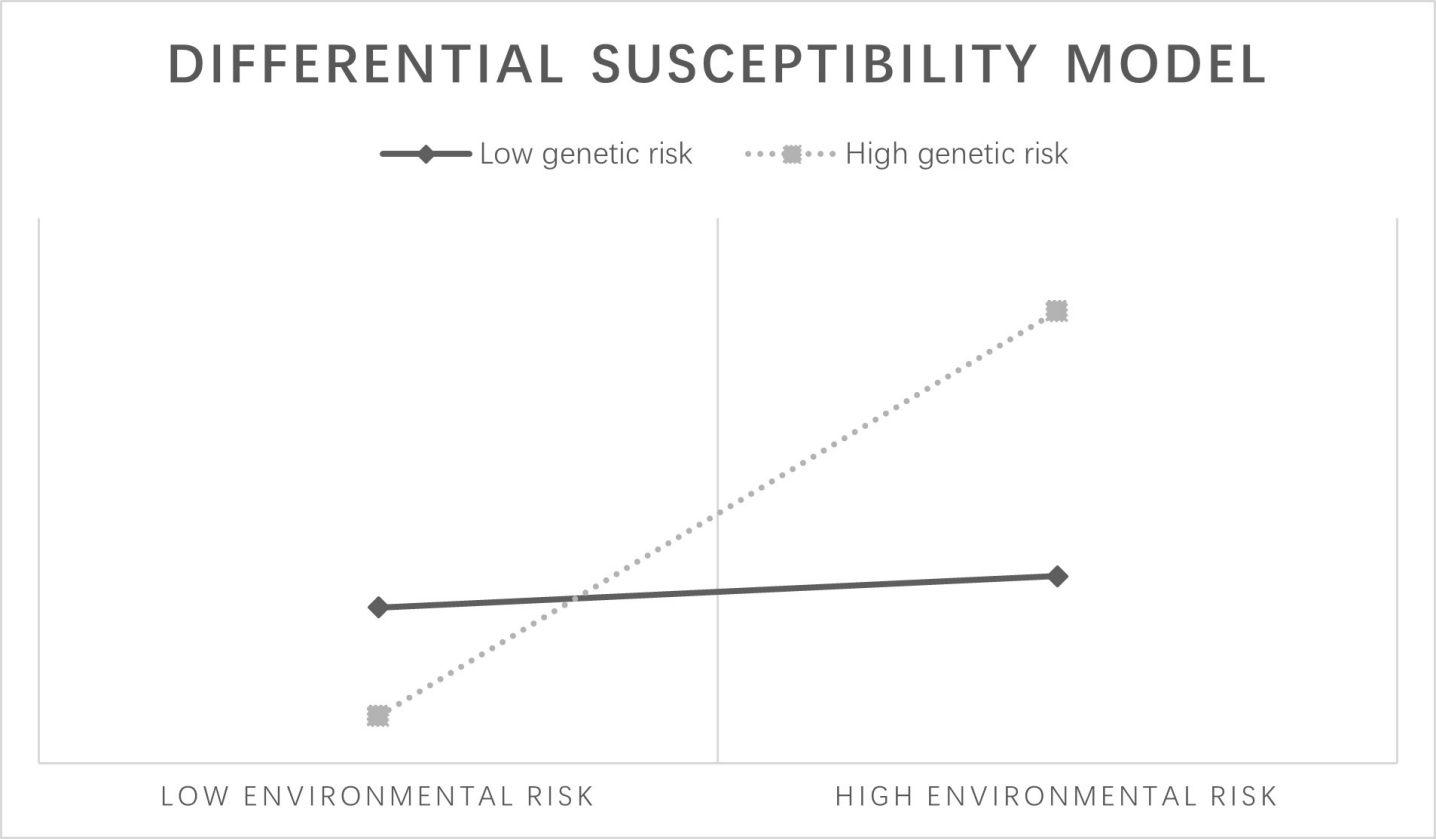
Social push model.

**Fig 4:**
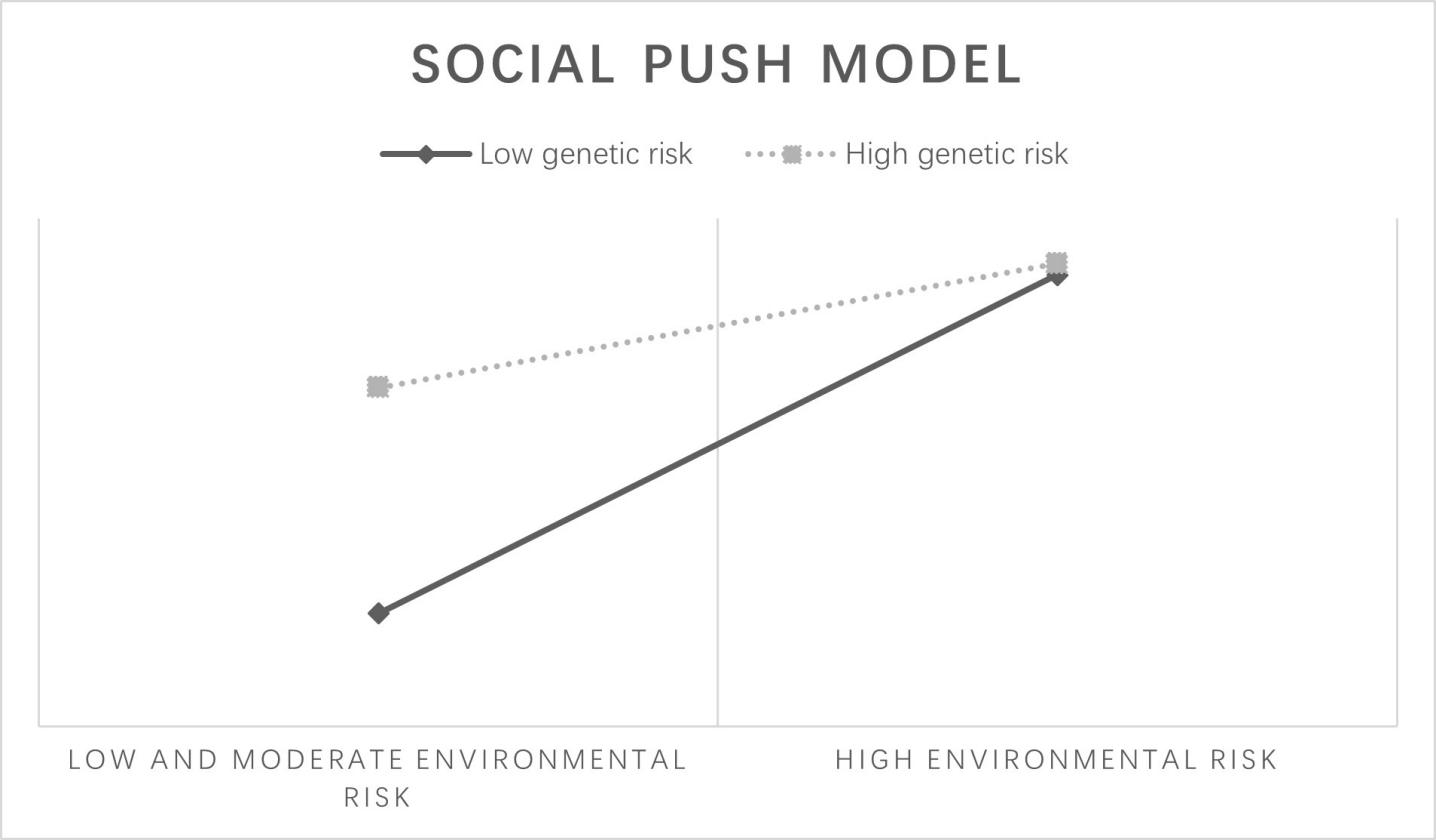
Swing model.

The other two models are less commonly observed in substance use behavior studies. The “social push” model (Fig 3) [23] implies that genetic factors have a strong effect at low or medium levels of environmental risk, whereas genetic risk has a lesser impact in high-risk environments perhaps because social influences dominate in extreme environments. The “swing” model (Fig 4)[24] suggests that individuals with medium genetic risk are most likely to be influenced by environmental factors to develop health risk behavior than individuals at the extremes with a low and high propensity.

Two systematic reviews have carefully considered G×E interactions in substance use and misuse [5, 14]. Pasman et al. [5] reviewed genome-wide association studies (GWAS), candidate gene score (CGS), and haplotype methods, showing that under some analytical frameworks a G×E effect can be viewed as weak. In a separate analysis approach, Do and Maes [14] found that 13 of 16 studies (81.2%) using either “candidate genes” or twin study, providing more evidence for significant G×E effects in patterns of maladaptive substance use. Specifically, Do and Maes highlighted the examination of nicotine use, through smoking studies. The analyses provided did not include an interpretative focus on the role of certain genetic or environmental factors [14]. Pasman et al. [5] focused on polygenic G×E studies, which may cause difficulty in investigating the exact genetic pathway or mechanism of the development of substance use behavior. For instance, GWAS, which can test thousands of genetic variants of many individuals to identify novel variant–trait association, may lead to discovery of new biological mechanisms. However, it carries the statistical burden of multiple testing, and sometimes only explains a modest fraction of heritability [25]. In addition, GWAS cannot directly determine causality when the mapping of most association signals occurs in non-coding regions of DNA, which makes biological interpretations of causality somewhat challenging, since these regions of DNA are directly engaged by forces determined by the cellular ‘environment’ [25].

In contrast, the candidate gene approach has been adopted by many studies in studying G×E effects of various unfavorable behavior such as antisocial behavior [26], gambling [27], and drug abuse [28]. The candidate gene method enables us to comprehensively understand and determine the exact neurological pathway of substance use behavior by classifying them into dopaminergic, serotoninergic, or other pathways. Although the candidate gene method is useful and economical to reveal genetic architecture of complex traits, it is restricted to existing knowledge and presumptions [29] and inconsistent findings in previous studies make it challenging to create a consistent picture of the G×E effect in certain behavior.

This study aims to provide an additional explanation or overview of potential GxE interaction effects on maladaptive patterns of substance use behaviors using, specifically, a candidate gene approach. We fit the studies into different theoretical G×E models and examine whether distinct G×E patterns exist for different races, genders, and types of substances. Our approach extends the previous work by further exploring the role of specific genetic and environmental factors in the development of substance use behavior.

## Methods

This review was in accordance with the PRISMA guideline [30].

### Search strategy

Literature searches were conducted in PubMed, psycINFO, and Google Scholar. Relevant peer-reviewed articles were included based on titles and abstracts. Keywords included substance use and gene–environment interaction; the detailed search formula and keywords are shown in S1 Table. In addition, we searched reference lists of published studies, meta-analyses, and review articles for more eligible articles. The last search took place on March 1st, 2022. Article screening was performed by two trained research staff using the same formula.

### Study eligibility

**Fig 5:**
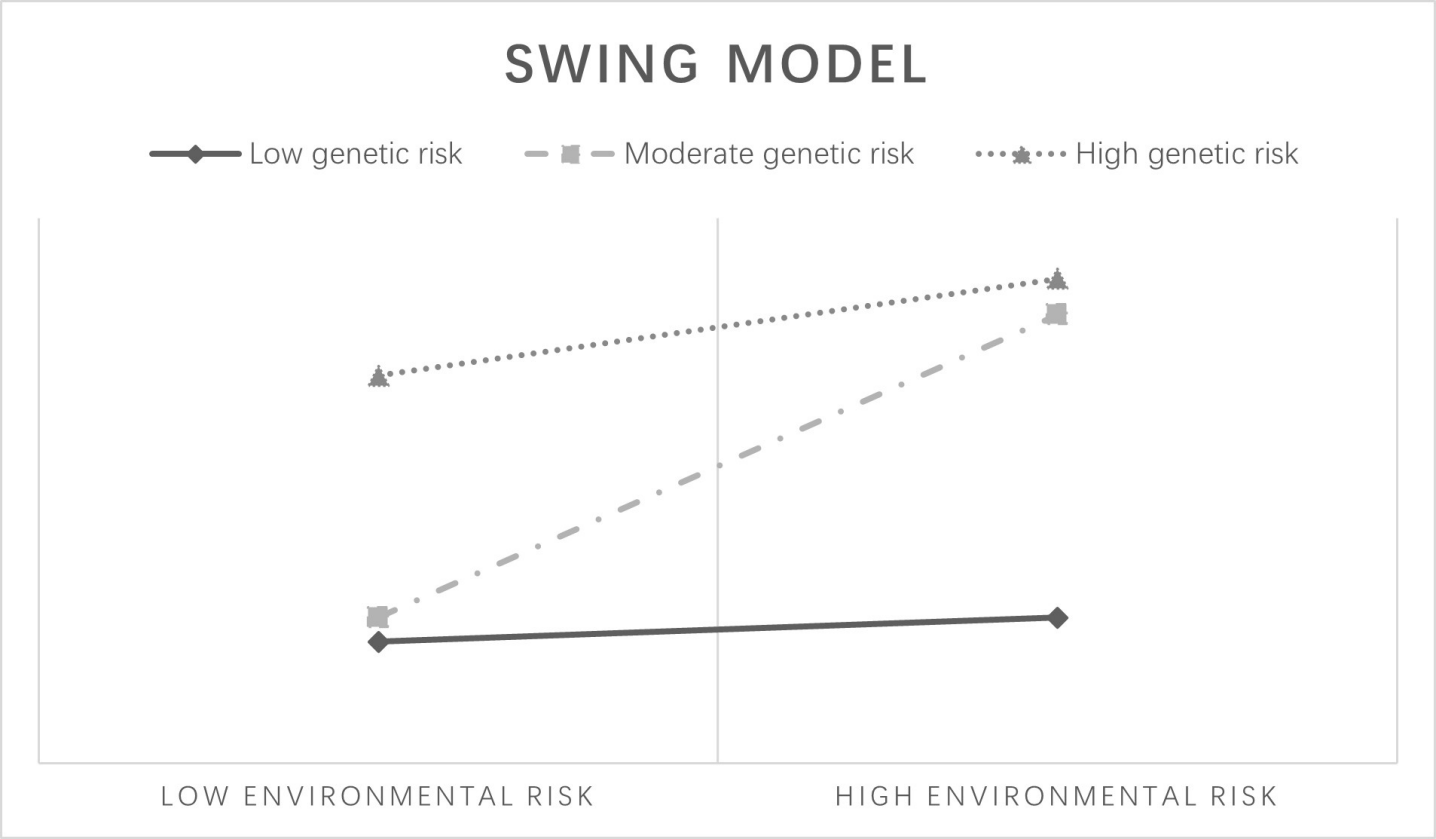
Flowchart of study selection for inclusion in this review.

Included studies should meet the following criteria: (1) human objects only; (2) the outcome of the study should be any kind of licit and illicit substance use; (3) studies focusing on candidate genes and environmental factors (e.g., psychosocial experiences and demographic characteristics); (4) the study was an original research report; (5) gene–environment interaction was calculated statistically in the study; (6) articles written in English (Fig 5).

### Data extraction

All data were extracted for the following variables: (1) article title, (2) 1st author, (3) study design, (4) study population, (5) data set used, (6) target gene, (7) environmental exposure and measure, (8) outcome measure, (9) demographics (e.g., age, gender) of the study population, (10) main findings, (11) statistical outcome, (12) *P* value on the G×E effect, (13) risk of bias, (14) rGE (genotype–environment correlation) status, (15) study power calculation, and (16) G×E effect pattern classification. Meta-analysis or publication bias assessment could not be conducted because of heterogeneity and statistical reporting differences. Data extraction was performed by two reviewers independently and cross-checked afterward.

### Result synthesis

The data were tabulated into two tables: Table 1 consisted of basic demographic data, wherein studies using the same dataset were identified and noted. Table 2 summarizes key information of studies, including target genes, environmental factors, outcomes, main findings, risk alleles, G×E patterns, wherein the main findings were summarized into brief sentences. The patterns were coded into four types: A (diathesis–stress model), B (differential susceptibility model), C (“social push” model), and D (“swing” model). The G×E patterns were directly obtained from the studies if they were explicitly stated in the article. Otherwise, the authors coded the pattern based on the G×E results. For example, if individuals with high genetic risk are more likely to engage in substance abuse when exposed to high-risk environments (e.g., maltreatment by parents), such a G×E result is consistent with the (A) diathesis-stress model.

**Table 1:** Basic demographics of the studies included.

1st Authors	Year	Study design	Age (mean)	Gender	Population	N (case)	Dataset
Bau [31]	2000	Case–control	41	All male	Caucasian males	229(115)	/
Madrid [21]	2001	Cross-sectional	37.8	All male	Adult Honduras males	309	/
Nilsson [32]	2005	Longitudinal	19–22	59.50% female	Swedish students	200	Sweden SALVe (Survey of Adolescent Life in Vestmanland)
Lerer^#^ [33]	2006	Case–control	20–30	All female	Jewish College students	390(242)	/
Dick [34]	2006	Longitudinal	NA	NA	Caucasians	1913	COGA (Collaborative Study on the Genetics of Alcoholism)
Kaufman [35]	2007	Longitudinal	12.5	58% female	Mixed American children	219	SAFE home
Covault* [36]	2007	Longitudinal	18.7±0.8	54% female	Non-Hispanic Caucasian college students	295	/
Nilsson [19]	2007	Longitudinal	19–22	All male	Swedish students	66	Sweden SALVe
Dick [17]	2007	Case–control	case: 37.1, control: 40.6	NA	NA	2127(1232)	COGA
Segman^#^ [37]	2007	Case–control	24	All female	Jewish College students	390(242)	/
Vanyukov [38]	2007	Longitudinal	18–19	All male	European American	144	CEDAR (Center for Education and Drug Abuse Research)
Blomeyer [18]	2008	Longitudinal	15	53.70% female	European descents	270	Germany Mannheim Study of Children at Risk
Gacek* [39]	2008	Longitudinal	18.65±0.86	54.4% female	Non-Hispanic Caucasian college students	351	/
Nilsson [40]	2008	Longitudinal	19–22	All female	Swedish students	114	Sweden SALVe
Ducci [41]	2008	Case–control	37.80±14.58	All female	Indian tribe adults	187(95)	/
Chen [42]	2009	Case–control	36.4	61.5% female, 54% in cases, 69.2% in controls	European American	2027(1032)	COGEND (Collaborative Genetic Study of Nicotine Dependence)
Du [43]	2009	Case–control	median = 37	23.7% female in controls, 18.9% female in cases	Mexican–Americans	703(365)	/
Laucht [44]	2009	Longitudinal	19	54% female	European descents	309	Germany Mannheim Study of Children at Risk
Schmid [45]	2010	Longitudinal	19	53.7% female	European descents	270	Germany Mannheim Study of Children at Risk
van der Zwaluw [46]	2010	Longitudinal	13.4	NA	Dutch adolescents	428	Netherland longitudinal family and health
Nelson [47]	2010	Case–control	42	78% female in cases, 46% in controls	European Australians	1128(156)	NAG (Nicotine Addiction Genetics) project
Enoch [22]	2010	Case–control	46 in cases, 34 in controls	All male	Adult African Americans	350(72)	SATP (Substance Abuse Treatment Program)
Ducci [48]	2011	Longitudinal	14 & 31	52% female	Newborns	4762	NFBC1966 (Northern Finland Birth Cohort of 1966)
Kranzler [49]	2011	Case–control	42 in African Americans (AAs), 38 in European Americans (EAs)	44% female in AAs, 42% female in EAs	African Americans, European Americans	3080 in total; EAs: 1211(330), AAs: 1869(634)	Yale-Penn (Yale-Pennsylvania)
Fletcher [50]	2012	Cross–sectional	42.83	52% female	Mixed Americans	7200	NHANES (National Health and Nutrition Health Examination III)
Xie [51]	2012	Correlational	38.1	41% female	European Americans	2206	Yale-Penn
Vaske [52]	2012	Longitudinal	19	51.8% female	Mixed American youths	2403	Add Health
Daw [53]	2013	Longitudinal	16.34	53% female	Mixed American youths	14560	Add Health
Perry [54]	2013	Correlational	40.56	56% female	Mixed Americans	2281	COGA
Miranda [55]	2013	Case–control	15.6	49% female	European ancestry adolescents	104(18)	/
Ray [56]	2013	Case–control	38.1±9.9 in case, 38.7±9.4 in controls	61% in cases, 70% in controls	European American adults	2533(1167)	SAGE (Study of Addiction: Genetics and Environment
Olsson [28]	2013	Longitudinal	24	NA	Australians	839	Victorian Adolescent Health Cohort
Hiemstra [57]	2014	Longitudinal	12.52 in study 1, 14.16 in study 2	53.1% female in study1, 52.1% female in study 2	Dutch adolescents, Dutch twins	991 in study1, 365 in study2	No dataset used in study 1; Netherland longitudinal family and health used in study 2
van der Zwaluw [58]	2014	Longitudinal	Followed from14.3 to 18.7	42.8% female	Dutch adolescents	299	Netherland longitudinal family and health
Handley [13]	2015	Case–control	16	55.8% male	Mixed American adolescents	326(179)	/
Rovaris [59]	2015	Longitudinal	28.9	All female	Brazilian women	139	/
Windle [60]	2016	Longitudinal	10–24	NA	African American children	2088	GEDID (Genes Environments and Development Integrated Dataset)
Bendre [61]	2018	Longitudinal	NA	All male	Swedish young males	53	/
Fite^$^ [62]	2019	Correlational	Overall sample: 18.95±1.19, males:19.14±1.25, females: 18.76±1.10	50.9% female	Students enrolled in undergraduate psychology courses at a large Midwestern university	470	/
Fite^$^ [63]	2019	Correlational	Overall sample: 18.95±1.19, males:19.14±1.25, females: 18.76±1.10	50.9% female	Students enrolled in undergraduate psychology courses at a large Midwestern university	470	/
Su [64]	2019	Longitudinal	12–21	53.3% female	Mixed American youths	13749	Add Health
Navarro-Mateu [65]	2019	Case–control	Controls: 48.42±16.14, cases: 42.30±10.27	55.1% female	European descents	673(142)	Controls were from Psychiatric Enquiry to General Population in Southeast Spain (PEGASUS)-Murcia project
Hendershot [66]	2020	Longitudinal	38	29.31% female	American adults	58	mHealth
Ossola [67]	2021	Case–control	55.53 in cases, 38,25 in controls	72.9% male in cases, 33.3% male in control	NA	107(59)	/

Studies denoted with

*, $, and # used the same subject population that is not from a dataset

**Table 2:** Summary of studies.

Authors	Genes	Environment	Outcomes	G×E effect (+/−) Main findings		G×E pattern (A: diathesis-stress; B: Differential susceptibility)	Risk allele/variant/haplotype
Nilsson [32]	*5-HTTLPR*	Family relations	Self-reported alcohol consumption per year; high intoxication frequency	*5-HTTLPR* × env +	Adolescents with the *LS variant* of the *5-HTTLPR* gene and with family relations being “neutral” or “bad” had a 12- to 14-fold increased risk for high intoxication frequency.	A	*LS variant*
Kaufman [35]	*5-HTTLPR*	Maltreatment	Self-reported alcohol use behavior	*5-HTTLPR* × env +	Subjects with *s-allele* and exposed to maltreatment are generally more likely to develop alcohol use. Heterozygous s/l genotype showed the greatest vulnerability to early alcohol use when exposing to maltreatment.	A	*S allele*
Covault* [36]	*5-HTTLPR*	Stressful life events	Self-reported drinking frequency; heavy drinking frequency; Drinking intention; Proportion of drug use days	*5-HTTLPR* × env +	For the *short (s) allele* carriers who experienced multiple negative life events in the prior year reported more frequent drinking and heavy drinking, stronger intentions to drink, and greater nonprescribed drug use. In individuals homozygous for the long (l) allele, drinking and drug use were unaffected by past-year negative life events.	A	*S allele*
Dick [17]	*5-HTTLPR*	Stressful life events	Alcohol dependence defined by DSM-III-R and Feighner criteria	*5-HTTLPR* × env -	No interaction	/	/
Laucht [44]	*5-HTTLPR*	①Early family adversity; ②Current stressful life events	Self-reported past 45-day drinks and binge drinking	*5-HTTLPR* × ① −; *5-HTTLPR* × ② +	When exposed to high psychosocial adversity, individuals with the LL genotype of *5-HTTLPR* exhibited more hazardous drinking than those carrying the *S allele* or those without exposure to adversity.	A	Homozygous *L allele*
Vaske [52]	*5-HTTLPR*	Childhood neglect	Alcohol use problem; Marijuana use frequency	*5-HTTLPR* × env +^e^	Childhood neglect only leads to significantly higher levels of marijuana use for females when they carry two copies of the *5-HTTLPR short allele*. Not in men or in alcohol use	A	Homozygous *S allele*
Daw [53]	*5-HTTLPR*	①School-level drinking; ②School- level smoking	Self-reported alcohol consumption; self-reported drinking frequency; self-reported cigarette smoked; Self-reported smoking frequency	*5-HTTLPR* × ①+^f^; *5-HTTLPR* × ②+^g^	More 5HTTLPR*S′ alleles are associated with more smoking and drinking behaviors when exposed to higher school drinking/smoking level.	B	*S allele*
Windle [60]	*5-HTTLPR*	①Residential instability; ②Neighborhood concentrated disadvantage	Self-reported substance use	*5-HTTLPR* × ① −^j^ *5-HTTLPR* × ② +^j^	Higher residential instability, in conjunction with the *short allele* of *5-HTTLPR*, was associated with the highest level and steepest gradient of growth in substance use across ages 10–24 years.	A	*S allele*
Su [64]	*5-HTTLPR*	Parenting quality	Self-reported alcohol use frequency and quantity	Male subjects: *5-HTTLPR* × env + Female subjects: *5-HTTLPR* × env −	Parenting quality was associated with lower likelihood of following the persistent heavy drinkers’ trajectory for males carrying the *short allele* of *5-HTTLPR*. Not significant in female.	A	*S allele*
Navarro-Mateu [65]	*5-HTTLPR*	Maltreatments and family negativities	Alcohol and drug abuse and/or dependence disorders based on DSM-IV	*5-HTTLPR* × env −	No interaction.	/	/
Nilsson [19]	*MAOA*	①Poor family relationship; ②Maltreatment/abuse	Past-year alcohol-related problem behavior	Male subjects: *MAOA* × ①*② +; *MAOA* × ① +; *MAOA* × ① +	Boys with the short (three-repeat) variant of the *MAO-A* gene, who had been maltreated/abused or came from families with poor relations, showed significantly higher scores of alcohol-related problems.	A	Low activity (three repeats)
Vanyukov [38]	*MAOA*	Parenting	Substance use disorder based on DSM-III	Male subjects: *MAOA* × env +	High activity 3.5- and four-repeat alleles are associated with lower risk of substance use disorder when parenting CAPIB score was entered as interaction term.	A	High activity (3.5 and four repeats)
Nilsson [40]	*MAOA*	①Unfovorable family relationship; ②Maltreatment/abuse	Past-year alcohol-related problem behavior; AUDIT index	Female subjects: *MAOA* × ① +; *MAOA* × ② -	Long (four repeats) variant of the *MAOA* gene in females interacted significantly with an unfavorable environment to increase the risk for high scores of alcohol-related problems	A	High activity (four repeats)
Bendre [61]	*MAOA*	Maltreatment	AUDIT score	Male subjects: *MAOA* × env+	Among participants who had been maltreated, *MAOA*-*uVNTR S allele* carriers displayed higher AUDIT scores than *L allele* carriers.	A	Low activity (two and three repeats)
Fite^$^ [62]	*MAOA*	①All child maltreatment; ②Physical abuse; ③Emotional abuse; ④Physical neglect	Self-reported cannabis use	Male subjects: *MAOA* × ③ +; *MAOA* × ② +^a^ Female subjects: *MAOA* × ② +; *MAOA* × ③ +^a^ All subjects: *MAOA* × ① +	For males, emotional abuse was positively associated with lifetime cannabis use at *MAOA*‐*L* but unrelated at *MAOA*‐*H*. Physical abuse was negatively associated with lifetime marijuana use for *MAOA*‐*H* males and unrelated for *MAOA*‐*L* males. Physical and emotional abuse were unrelated to lifetime cannabis use in *MAOA*-*L* female but increase cannabis use in *MAOA*-*H* females.	A	High activity (3.5 and four-repeat) allele for female. Low activity (two and three-repeat) allele for male
Fite^$^ [63]	*MAOA*	①Child maltreatment; ②Physical abuse; ③Emotional abuse	Self-reported polysubstance use	Male subjects: *MAOA* × ① −; *MAOA* × ② +; *MAOA* × ③ +; Female subjects: *MAOA* × ① +^a^	For *MAOA*-*L* males, emotional abuse was positively associated with the number of substances used. For *MAOA*-*H* males, physical abuse was statistically negatively associated with the number of substances used. For homozygous *MAOA*-*H* females, there was a statistically significant positive association between any maltreatment type, and physical abuse, and emotional abuse and number of substances used.	A	High activity (3.5–4 repeats)
Ducci [41]	*MAOA;* *MAOB*	Childhood sexual abuse	Alcoholism determined by CAGE based on DSM-3	Female subjects: *MAOA* × env +; *MAOB* × env −	Sexually abused female carrying homozygous low activity allele has higher risk of alcoholism; The G×E effect in *MAOB* genotype is not significant.	A	Low activity (2–3 repeats)
Bau [31]	*DRD2*	①Stress; ②Harm avoidance	Physiological alcohol dependence based on DSM-III-R	Male subjects: *DRD2* × ①+; *DRD2* × ②+	Patients with *DRD2 Taq A1 allele* with higher stress or higher harm avoidance are more likely to develop dependence.	A	*A1 allele*
Madrid [21]	*DRD2*	①Total stress score determined by HIS, occupational; ②Economical stress	Alcoholism score determined by MAST	Male subjects: *DRD2* × ①+; *DRD2* × ②+	*A1+* people have higher MAST score than *A1−* at a high-stress level, whereas they have lower MAST score at a low-stress level.	B	*A1 allele*
van der Zwaluw [46]	*DRD2*	Parental alcohol rule setting	Self-reported past week drinks	*DRD2* × env +	Adolescents with parents highly permissive toward alcohol consumption and carrying a genotype with the *DRD2 A1 (rs1800497T) allele*, used significantly more alcohol.	A	*A1 allele*
Olsson [28]	*DRD4*	Insecure attachment	Self-reported frequency of tobacco use; Frequency of cannabis use; Frequency of alcohol use	*DRD4* × env +^i^	7R+ alleles will intensify the effect of insecure attachments for cannabis use, no evidence of interaction in tobacco and alcohol use.	A	seven repeats
Segman^#^ [37]	*DAT1*	Traumatic life experience	Smoking initiation; Nicotine dependence measured by FTQ	Female subjects: *DAT1* × env +	Interaction between trauma and the *DAT1_E15þ274*—*DAT1_VNTR C-9* haplotype made a significant protective contribution to the model	A	*DAT1_E15þ274*—*DAT1_VNTR C-9*^*l*^
Hiemstra [57]	*DRD2;* *DRD4;* *DAT1*	①Maternal smoking; ②Paternal smoking; ③Friend’s smoking; ④Best friend’s smoking; ⑤Sibling smoking	Self-reported smoking	Study1: *DRD2/DRD4/DAT1* × env − Study2: *DRD2/DRD4/DAT1* × env −	No interaction.	/	/
Blomeyer [18]	*CRHR1*	Negative life events	Current Drinking; Max drinks/occasion; Mean drinks/month; Lifetime heavy drinking	*CRHR1(rs1876831)* × env +; *CRHR1*(rs242938*)* × env −	Adolescents homozygous for the *C allel*e of *rs1876831* drank higher maximum amounts of alcohol per occasion and had greater lifetime rates of heavy drinking in relation to negative life events than individuals carrying the *T allele*.	A	Homozygous *C allele* of *rs1876831*
Schmid [45]	*CRHR1*	Stressful life events	Age at first drink; Number of drinks; Max drinks/occasion; Drinking days; Binge days	*CRHR1 rs1876831* × env +^b^; *CRHR1 rs242938* × env +^c^	Homozygotes of the *rs1876831 C allele* as well as carriers of the *rs242938 A allele*, when exposed to stress, exhibited significantly higher drinking activity than carriers of other alleles.	A	Homozygous *rs1876831 C allele*; Homozygous *rs242938 A allele*
Nelson [47]	*CRHR1*	Childhood sexual abuse	Alcohol use; Alcohol dependence according to DSM-IV	*CRHR1* × env +	*CRHR1 H2* haplotype protects against childhood sexual abuse-associated effects on alcohol dependence risk.	A	*H2* ^*l*^
Kranzler [49]	*CRHR1*	Childhood adverse events	Alcohol dependence based on DSM-IV	*CRHR1* × env −	No interaction.	/	/
Ray [56]	*CRHR1*	Trauma exposure	Alcohol dependence based on DSM-IV	*CRHR1* × env +^h^	In block 1, *H1* haplotype is a protective factor in individuals exposed to trauma, in block 2, *H1* or *H7* are also protective factors.	A	Block 1: *H1*; Block 2: *H1*, H7^l^^,^^m^
Dick [17]	*GABRA2*	Marital status	Lifetime alcohol dependence based on DSM-4	*GABRA2* × env +	There was no difference in rates of alcohol dependence by genotype. However, among married individuals, high-risk genotype carriers are more likely to develop alcohol dependence.	A	Homozygous *A allele of rs279871*
Enoch [22]	*GABRA2*	Childhood trauma	Alcohol dependence based on DSM-IV; Substance dependence based on DSM-IV	Male subjects: *GABRA2* × env +^d^	*GABRA2 rs11503014 11/12* genotype is associated with increased risk when exposed to higher childhood adversity while associated with decreased risk when the adversity is low.	B	*rs11503014 11/12*
Perry [54]	*GABRA2*	Daily life events	Lifetime alcohol dependence based on DSM-4	Male subjects: *GABRA2* × env +	For men with the high-risk genotype, additional positive daily experiences were associated with a significant decrease in risk for alcohol dependence	B	Homozygous *A allele of rs279871*
Miranda [55]	*OPRM1*	①Deviant peer affiliation; ②Parental monitoring	Alcohol use disorder according to DSM-IV	*OPRM1* × ①+; *OPRM1* × ②+	*G allele* carriers with high levels of deviant peer affiliation or lower levels of parental monitoring had the greatest likelihood of developing alcohol use disorder.	A	*G allele*
Hendershot [66]	*OPRM1*	Naltrexone adherence	Self-reported daily drinking and craving level	*OPRM1* × env +^k^	People with *AG/GG* gene type are more likely to drink more in those low adherence population.	A	*AG/GG*
Chen [42]	*CHRNA5;* *CHRNA3*	Parent monitoring	Nicotine dependence	*CHRNA5(rs16969968)* × env +; *CHRNA3(rs3743078)* × env -	The risk for nicotine dependence increased with the risk genotype of SNP (*rs16969968*) when combined with lowest quartile parent monitoring. In contrast, there was no evidence of an interaction between SNP (*rs3743078*) and parent monitoring.	A	*rs16969968 (AA) *
Fletcher [50]	*CHRNA6*	State-level tobacco tax rate	Serum nicotine level; self-reported smoking	*CHRNA6* × env +	People with *CHRNA6 GG* subtype tend to smoke less when tax is high.	A	*rs2304297 GG*
Xie [51]	*CHRNA5*	Childhood adversity	Alcohol dependence based on DSM-IV	Male subjects: *CHRNA5 rs16969968* × env +	*CHRNA5 rs16969968 AA* genotype has the highest risk of developing ND if this man experienced childhood adversity. No significant interaction in women was found.	A	*rs16969968 AA*
van der Zwaluw [58]	*DRD2; OPRM1*	Parental rule setting	Self-reported past week drinks	*OPRM1* × env +; *DRD2* × env −	The alcohol use of *OPRM1 G allele* carriers was affected by parental rule-setting, more rules lead to less alcohol use, while *A/A* genotype carriers remained largely unaffected by parental rules.	A	*OPRM1 G*
Du [43]	*5-HTTLPR;* *OPRM1;* *DRD2*	①Current marital status; ②Education	Alcohol use disorder severity	*OPRM1* × ② +^a^	*OPRM1 A/A* being a high-risk factor of the alcohol use disorder in people with education≤12 years.	A	*OPRM1 A/A*
Ducci [48]	*CHRNA5-CHRNA3-CHRNB4;* *TTC12-ANKK1-DRD2*	①Novelty seeking; ②Maternal smoking	Self-reported smoking at 14 and 31 years old	*CHRNA3 rs1051730* × ② +^a^	Among offspring of smoking mothers, *CHRNA3-rs1051730* was associated with significantly reduced risk of being occasional smokers than non-smokers at age 14 years. No interaction in the other genotypes or environmental factors.	A	*CHRNA3 rs1051730A allele* ^l^
Ossola [67]	*5-HTTLPR;* *DRD2*	①Socio-demographic variables; ②Adverse childhood experiences; ③Psychopathology symptoms	Alcohol use disorder based on DSM-5	*5-HTTPLR* × any env −; *DRD2* × any env −	No interaction	/	/
Lerer^#^ [33]	*HTR6;* *HTR1B*	Traumatic life experience	Smoking initiation; Nicotine dependence measured by FTQ	Female subjects: *HTR6* × env +	The interaction of *HTR6-C276T* genotype and lifetime traumatic experience contributed strongly to the risk of smoking initiation and nicotine dependence.	A	*HTR6-C276T*
Gacek* [39]	*TPH2;* *TPH1;* *5-HTTLPR*	Negative life events	Self-reported alcohol consumption; Drinking frequency	TPH1 × env −; TPH2 × env −; *5-HTTLPR* × env -	No interaction	/	/
Handley [13]	*FKBP5*	Childhood maltreatment	Marijuana use discovered by using Diagnostic Interview Schedule for Children (DISC)	FKBP5 × env +	Child maltreatment predicted more marijuana dependence symptoms among adolescents with 1 or 2 copies of the *CATT* haplotype only.	A	1 or 2 copies of the CATT haplotype
Rovaris [59]	*NR3C1;* *NR3C2*	①Physical neglect; ②Emotional neglect; ③Physical abuse; ④Sexual abuse; ⑤Emotional abuse	Cocaine use	Female subjects: *NR3C2 rs5522* × ① +; *NR3C2 rs5522* × ② +; *NR3C2 rs5522* × ③ −; *NR3C2 rs5522* × ④ −; *NR3C2 rs5522* × ⑤ −; *NR3C1 rs6198* × ① −^a^	*NR3C2 rs5522-Val* can increase susceptibility to cocaine in physical neglect and emotional neglect population. *NR3C1 rs6198-G* is not associated with physical neglect.	A	*NR3C2 rs5522-Val*

Studies denoted with

*, $, and # used the same subject population that is not from a dataset

^a^ The rest of the G×E combinations are not statistically significant.

^b^ The G×E effect is significant in outcomes of age at first drink/number of drinks/max drinks on an occasion but not in drinking days and binge days.

^c^ The G×E effect is significant in outcomes of binge days or number of drinks but not in drinking days/age at first drink/max drinks on an occasion.

^d^ The G×E effect is only significant in cocaine use.

^e^ Marijuana use frequency only; the G×E effect on alcohol use was not reported.

^f^ In full sample, the G×E effect is statistically significant only in the number of drinks consumed but not in drinking frequency. In sibling sample, the G×E effect is statistically significant in the number of drinks consumed and drinking frequency.

^g^ In full sample, the G×E effect is statistically significant in the number of cigarettes consumed and smoking frequency. In sibling sample, the G×E effect is statistically significant only in the number of cigarettes consumed but not in smoking frequency.

^h^ Only *H1* haplotype in block 1 and *H1*, *H7* haplotype in block 2 have statistically significant G×E effect, and the rest of the haplotypes are not significant.

^i^ The G×E effect is only significant in cannabis use but not in tobacco or alcohol use.

^j^ By adding age as a covariate, *5-HTTLPR* X residential stability turns significant, whereas *5-HTTLPR* X neighborhood disadvantage turns non-significant.

^k^ The G×E effect in the outcome of drinks per day is statistically significant but not in same-day craving.

^l^ Allele/variant works as a protective factor

^m^ Different from the common classification of *H1* or *H2* haplotype, *H1* in block 1 stands for *rs4792886[G]*+*rs110402[C]*+*rs242924[C]*; *H1* in block 2 stands *for rs242942[G]*+*rs3785877[G]*+*rs171440[C]*+*rs242939[A]*+*rs4566211[A]*+*rs242936[C]*+*rs17762954[T]*+*rs173365[T]*+*rs1396862[T]*+*rs17763086[G]*+*rs17763104[G]*+*rs16940665[C]+rs17689918[A]+rs17689966[G]+rs1876829[G]*; *H7* in block 2 stands for *rs242942[A]+rs3785877[A]+rs171440[C]+rs242939[A]+rs4566211[G]+ rs242936[C]+rs17762954[C]+rs173365[T]+rs1396862[C]+rs17763086[T]+rs17763104[G]+rs16940665[T]+rs17689918[G]+rs17689966[G]+rs1876829[A]*.

## Results

The study selection process is summarized in the flowchart shown in Fig 1. A total of 44 articles were included in this systematic review. We will discuss the results of the review based on individual gene types, and their key features and G×E findings will be summarized. Moreover, pattern classification will be reported and compared across gene types, genders, and races.

### Study description

Key demographic data of each study are summarized in Table 1. The major features and G×E findings are summarized in Table 2.

### Gene-specific results

#### 5-HTTLPR

The serotonin transporter gene-linked polymorphic region (*5-HTTLPR*) is located in the promoter region of the *SLC6A4* gene, which codes for presynaptic serotonin transporters responsible for serotonin reuptake [7]. The *5-HTTLPR* starts at 28,521,337 base pairs from the pter and consists of a 20–23 base pair repeat sequence. The *short (S) allele* of *5-HTTLPR* has lower transcriptional efficiency compared with the *long (L) allele*; thus, people carrying *S-allele* have a higher and unstable concentration of serotonin in the synaptic cleft [7]. Serotonin as a neurotransmitter is involved in the physiological processes of mood [68]. Studies have found that *S-allele* is related to an increased risk of mental health disorders [69, 70], including polysubstance use [71].

There are 13 studies on *5-HTTLPR* × environment interaction (Table 2), where environment factors included socio-demographic variables, childhood adversities, and negative life events. Eight studies reported a significant G×E effect, whereas the remaining five studies showed no significant interaction. Among studies with significant G×E interactions, seven were consistent with the diathesis-stress model [32, 35, 36, 44, 52, 60, 64], whereas one study was consistent with the differential susceptibility model [53]. Six studies suggested *S-allele* as the risk allele [35, 36, 52, 53, 60, 64]; one study indicated homozygous *L-allele* as the risk allele [44], and one advocated *LS variant* as the risk allele [32].

Other than age, no notable differences were found among the five studies [17, 39, 43, 65, 67] with non-significant results regarding study design, environment factor selection, and gender. Four [17, 43, 65, 67] out of the five studies had a mean age over 35, whereas the mean age of the eight studies with significant G×E results had a mean age below 25 [32, 35, 36, 44, 52, 53, 60, 64]. It may suggest that substance use behavior can be affected by age, possibly due to complicated environmental exposure or different social norms between generations.

#### MAOA

*MAOA* codes are involved in the degradation of brain monoamine transmitters such as serotonin, dopamine, and norepinephrine. The enzyme plays a role in stress response and addiction pathogenesis. The *MAOA* gene is located on the X-chromosome (Xp11.4–p11.23) [62]. The best characterized genetic variants of *MAOA* are related to an upstream Variable Number Tandem Repeat (*uVNTR*), comprising a 30-basepair repeat sequence present in 2, 2.5, 3, 3.5, 4, 5, or 6 copies located in the gene promoter [72, 73]. Alleles harboring two and three repeats are classified as “low-activity variant,” whereas three and four repeats are referred to as “high-activity variant.” The rest of the repeats, including 2.5, five, and six repeats, are not studied because of lack of evidence of their exact functions.

Studies have revealed the association of *MAOA* and substance use behavior [74, 75], particularly in male subjects [76]. We found seven studies on *MAOA* × environment interaction (Table 2), with six focusing on childhood adversities [19, 40, 41, 61–63] and one focusing on parenting quality [38]. All the studies reported significant G×E effect and were consistent with the diathesis–stress model However, the risk allele varied among different studies. Three studies reported low-activity allele (2–3 repeats) as the risk allele [19, 41, 61]; three studies reported high-activity allele (3.5–4 repeats) as the risk allele [38, 40, 63].

In male subjects, of the five studies, four reported low-activity allele as the risk allele. In female subjects, three out of four studies advocated high-activity allele as the risk allele in contrast to male subjects. Although the mechanism of the interaction between sex and *MAOA* remains unclear, *MAOA* is an X-linked polymorphism, whereas X-inactivation of female might increase the variability of X-linked gene expression [77]. Aside from gene expression, androgen might also explain the sex-dimorphic characteristic because testosterone may interact with *MAOA uVNTR* variants to predispose aggression and risk-taking behavior [78, 79].

#### DRD2 DRD4 DAT1

These three genes are all related to the dopaminergic system in the brain. The *DRD2* gene codes for the D2 subtype of the dopamine receptors. It is located on chromosome 11 q22–q23 [80]. The *A1-allele* for the *TaqIA* polymorphism (*rs1800497*) is associated with significantly reduced amount of D2 receptors in the brain compared with the *A2-allele* [81], which might decrease reactivity toward dopamine signals. A meta-analysis of the relationship between D2 dopamine receptor gene and alcoholism reported that alcoholics had a higher prevalence of the *A1 allele* than controls [82], whereas another meta-analysis of dopaminergic receptors indicated a weak association between DRD4, DAT gene and smoking initiation [83].

The *DRD4* gene codes for the D4 subtype of dopamine receptors. It is located on chromosome 11p15 [84]. Variable number tandem repeat (*VNTR*) in exon III of the *DRD4* gene is particularly noteworthy, which comprises a 48 nucleotide repeat sequence and 2–11 repeat units (2R-11R) [28]. Alleles with 2–5 repeats are commonly identified as short, whereas alleles with 6–10 repeats are referred to as long allele. Long alleles, particularly the 7R allele, has a blunted ability to reduce cAMP levels, thereby suppressing the gene expression compared with short alleles[85], whereas the low amount of dopamine receptors has been related to substance abuse [86].

The *DAT1* gene (*SLC6A3*) mediates the presynaptic reuptake of dopamine. It is located on chromosome 5p15.32, and the polymorphism *VNTR* comprising a 480 base pair repeat sequence is located on the 3′ untranslated region of the gene starting 1,392,905 base pairs from pter [87]. The 9R allele is associated with the low expression of the gene, which lowers the levels of the transporter protein in the brain [88], leading to higher brain levels of dopamine.

A total of nine studies focused on the interaction between these dopaminergic genes and environmental factors, and seven studies included *DRD2* [21, 31, 43, 46, 57, 58, 67]. In addition, two studies included *DRD4* [28, 57], and two studies investigated *DAT1* [37, 57]. The environmental factors included childhood adversities, psychological factors, family factors, and socio-demographic factors. Five out of nine studies reported at least one significant G×E interaction on substance abuse [21, 28, 31, 37, 46].

Seven studies examined *DRD2* × environment interaction (Table 2). Three out of seven studies reported a significant G×E effect of the *DRD2* gene [21, 31, 46], three of which supported *A1 allele* as the risk allele [21, 31, 46]. Among studies with significant associations, two were consistent with the diathesis-stress model [31, 46], and one showed support for the differential susceptibility model [21].

There were two studies on *DRD4* × environment interaction [28, 57] (Table 2). One study reported a significant interaction between *DRD4* (seven repeats allele) and insecure attachment on cannabis use, which was consistent with the diathesis-stress model [28]. Among the two studies on *DAT1* × environment interaction [37, 57] (Table 2), one reported a significant interaction between *DAT1_E15þ274*—*DAT1_VNTR C-9* and traumatic life experience in smoke initiation and nicotine dependence. The G×E pattern showed supported for the diathesis-stress model.

The G×E effect of the *DRD2* gene remains controversial, and only some early research found a significant interaction between *DRD2* and environmental factors. Two out of the three significant studies used male subjects, whereas four non-significant ones used male and female subjects. Considering that many studies favored the association of *DRD2* TaqIA with substance addiction in male subjects [89, 90], differential selection might be involved in this controversial result. Regarding the differences in environmental factors selection, studies using socio-demographic variables generated a different result from those studies that used stress as the environmental factor. This finding could be due to high neural reactivity toward emotional stress in subjects carrying *A1 allele* [91]. For *DRD4* and *DAT1* genes, the results were inconclusive because of the limited number of studies. Gender, environmental exposure selection, or outcome selection may also be involved.

#### CRHR1

The *CRHR1* gene codes for corticotropin-releasing hormone receptor in the pituitary gland, which is located in the chromosome 17q21.31 [92]. The gene is approximately 51.55 kb in length, which is contained within a 900 kb inversion polymorphism [93], resulting in *H1* or *H2* haplotypes. The homozygous *H1* haplotype is more prevalent among African ancestry, whereas the *H2* haplotype accounts for 20% in individuals from European ancestry [93]. Treutlein et al. [94] studied the association between 14 haplotypes and alcohol use behavior and revealed that two SNPs, namely, *rs242938* and *rs1876831*, may be directly associated with the alcohol use behavior.

Five studies have investigated *CRHR1* × environment interaction [18, 45, 47, 49, 56] The environmental factors included negative life events and childhood adversities. Four out of five studies reported a significant G×E effect on substance use behavior [18, 45, 47, 56]. Two studies showed that homozygous *rs1876831 C allele* is the risk allele [18, 45], whereas one indicated *rs242938 A allele* as the risk allele [45]. Nelson et al. [47] reported *H2* haplotype as the protective haplotype, whereas Ray et al. [56] reported that *H1* in block 1 and *H1/H7* in block 2 were the protective haplotypes for alcohol dependence. With regard to the G×E pattern, all four studies with a significant G×E effect showed support for the diathesis-stress pattern.

Several studies found no significant interaction between *TAT* haplotype (*rs7209436*, *rs242924*, *rs110402*) and environmental factors [47, 49] One study [56] even found that *rs110402* and *rs242924* SNPs in *TAT* haplotype had a protective effect on trauma-exposed individuals but increased risk for alcohol dependence in individuals who had not experienced adulthood trauma.

#### OPRM1

The *OPRM1* gene codes for a protein known as the mu (μ) opioid receptor, which is part of the endogenous opioid system for regulating pain, reward, and addictive behavior. It is located on chromosome 6q24–q25 [95]. The *A118G variant* (*rs1799971*) is located in the exon 1 of the *OPRM2* gene, which has received the most attention because the variant moderates responses to psychoactive drugs such as alcohol [96]. The *G allele* has an amino acid change from asparagine to aspartic acid, and the Asp-containing receptor has three times higher affinity to β-endorphin, which can enhance positive feelings and craving following alcohol use [97, 98].

Four studies examined *OPRM1* × environment interaction [43, 55, 58, 66], and environmental factors included family factors, socio-demographic factors, and medication adherence. All four studies reported a significant G×E effect, which were consistent with the diathesis-stress model. Besides, three studies implicated *G allele* as the risk allele [55, 58, 66], whereas one study indicated homogenous *A allele* as the risk factor for alcohol use [43].

Du et al. [43] indicated that *OPRM1 A/A* instead of *G allele*, is a risk factor in subjects with education less than 12 years. This conflicting result may be due to environmental exposure selection. Du et al. [43] used education as the environmental exposure, whereas the other three studies used either family factors or medication adherence [55, 58, 66]. The *OPRM1* gene might also be a reason for the conflicting results because no association between *G allele* and alcohol use behavior was found in some studies [99, 100], whereas *A allele* was advocated as the risk allele in some other studies [101].

#### GABRA2

The *GABRA2* gene codes for Gamma-aminobutyric acid (GABA) A receptor, alpha 2 subunit. The *GABA-A* receptor is a ligand-gated chloride activated by inhibitory neurotransmitter GABA and psychoactive drugs such as benzodiazepines. It is located on chromosome 4p12 [102]. The gene can be suitable for examining genetic influence in the disinhibition pathway because of its role in impulsivity [103], and *GABRA2* might be associated with alcohol or illicit drug use behavior [104–106].

We found three studies on *GABRA2* × environment interaction [17, 22, 54]. The environmental exposures included marital status, childhood adversity, and daily positive life events. All three studies reported a significant G×E effect, of which two implicated homozygous *A allele* of *rs279871* as the risk allele and one indicated *rs11503014 11/12* genotype as the risk factor. Regarding the G×E effect, two studies reported a differential susceptibility pattern [22, 54], and one study reported a diathesis-stress pattern [17].

Enoch et al. [22] found that *rs11503014* was associated with heroin addiction, and it had a significant interaction with childhood trauma on cocaine use. Homozygote 11 and heterozygote 12 increased the risk with exposure to higher childhood adversity but decreased the risk when the adversity was low in cocaine dependence. The other two studies reported *rs279871 AA* genotype as the risk factor for alcohol dependence, the G×E pattern reported by Dick et al. [17] was consistent with the diathesis-stress model while the other two studies showed support for the differential susceptibility pattern [22, 54].

#### CHRNA3 CHRNA5 CHRNA6

These *CHRN* genes code for nicotinic acetylcholine receptor subunits alpha three, five, and six. These genes are located on chromosome 15q25.1 and are associated with smoking behavior in some large GWAS [107–109]. The associations have been found between the *rs1051730 A allele* of *CHRNA3/*the *rs16969968 A allele* of *CHRNA5* and nicotine dependence [110–113]. With regard to *CHRNA6*, the *rs2304297* variant was found to be associated with [114] tobacco use behavior [115, 116].

Four studies have examined the interaction between *CHRNA gene* and various environmental exposure, such as family factors and childhood adversity. Two studies reported the significant G×E effect of the *CHRNA5* gene with *rs16969968 AA* as the risk genotype; one study reported the significant effect of *CHRNA3* with *rs1051730 A* allele as the protective allele, and one study reported the significant effect of the *CHRNA6* gene with *rs2304297 GG* genotype as the risk genotype. The G×E patterns of all four studies were consistent with the diathesis-stress model.

Chen et al. [42] and Ducci et al. [48] studied two similar genes: *CHRNA3* and *CHRNA5*; Chen favored the G×E effect of *CHRNA5*, and Ducci favored that of *CHRNA3*. The difference could be due to the differential selection of the environmental and demographic variables. In Ducci et al. [48] study, G×E was significant among 14-year-old Finnish subjects, whereas Chen et al. [42] studied European American subjects with a mean age of 36.4 years.

For the three studies focusing on *CHRNA5*, all of them studied SNP *rs16969968* [42, 48, 51]. However, the G×E effect of this variant remained mixed. While Ducci et al. [48] reported no significant G×E effect, Xie et al. [51] reported a significant interaction between *CHRNA5* and childhood adversity on alcohol dependence in male subjects.

#### Other genes

Other less commonly studied genes, such as the *HTR* gene, which codes for the 5-hydroxytryptamine receptor, were also associated with alcohol and drug abuse behavior [117]. According to Lerer et al. [33], the *HTR6* gene, which is located on chromosome 1p35–p36, had a significant Interaction with traumatic life experience on smoking initiation, and *C267T* genotype was indicated as the risk genotype.

*TPH* genes, coding for tryptophan hydroxylase, were also involved in the serotonin neurotransmission pathway, which might have a potential influence on substance dependence development [39], but the previous study failed to find any association between them [118]. *TPH1* and *TPH2* gene, are located on chromosome 11p15.3–14 [119] and chromosome 12q21 [120], respectively. According to Gacek et al. [39], there was no interaction between TPH1 or TPH2 gene and negative life events on drinking behavior.

*NR3C2* gene codes for mineralocorticoid receptor, and *rs5522* is the most studied SNP. The *rs5522-Bal allele*, located on chromosome 4q31.1 [121], has been associated with altered reward learning [122] and tobacco use [123]. In Rovaris et al. [59], *NR3C2 rs5522-Val* allele exhibited a significant interaction with physical and emotional neglect on cocaine use.

#### Significance of the G×E effect

Of the 44 studies, 38 (86.36%) reported at least one significant G×E effect (see S2 Table). Among 32 studies with alcohol-related outcomes, 25 (78.13%) reported a significant G×E effect. Six out of eight (75%) studies on smoking behavior showed a significant G×E effect. With regard to marijuana, cocaine, and polysubstance use, these outcomes were less studied by researchers with only four studies on marijuana, three on polysubstance, and one on cocaine use, all of which reported a significant G×E effect.

European ancestry is the most studied race, with 23 studies looking into the G×E effect in this population, 18 of which (78.26%) reported a significant G×E effect. Three studies examined the G×E effect in African American population, two of which (66.67%) reported at least one significant G×E effect. Three studies focused on the G×E effect in Latino subjects, all of which (100%) reported at least one significant G×E effect.

In total, 15 studies reported the G×E effect separately by gender. Particularly, 10 studies reported the G×E effect in male subjects [19, 21, 22, 31, 38, 54, 61–64], all of which (100%) exhibited a significant G×E effect. Among 10 studies report female-specific results [33, 37, 40, 41, 51, 54, 59, 62–64], seven (70%) reported a significant interaction effect. The G×E effect of substance use behavior seems to exhibit sex dimorphism and the effect was more prominent in males.

#### G×E patterns

The G×E patterns are summarized in the column of “G×E pattern” in Table 2. Among the 38 studies, 34 significant G×E findings followed the diathesis–stress pattern [16], which suggests that individuals with high genetical risk likely experience higher levels of adverse outcome when exposed to a risky environment.

The diathesis-stress pattern was reported in all 18 studies focusing on subjects with European ancestry. In contrast, among two studies focusing on African-American subjects, one supported the diathesis-stress model and the other was consistent with differential susceptibility. For Latino subjects, two diathesis-stress and one differential susceptibility pattern were reported. The differential susceptibility G×E pattern seems to occur more often among African–Americans and Latinos than in European ancestry subjects. However, this finding must be further assessed with caution because of the limited number of studies and data on these two ethnic minorities.

The G×E pattern exhibited sex dimorphism. Of the 10 studies that reported a significant G×E pattern in male subjects, eight (80%) reported a diathesis-stress G×E pattern, and two (20%) reported a differential susceptibility pattern. By contrast, in female subjects, all significant studies (7 out of 7) reported a diathesis-stress G×E pattern.

## Discussion

In our systematic review, we reviewed 44 studies on G×E effect in various substance use behaviors with mixed results. The significant G×E effect was reported in 38 studies, 34 of which were consistent with the diathesis-stress pattern and four showed support for the differential susceptibility pattern.

### Mechanism and theory interpretation

The mechanisms behind these four G×E models appear to settle within three often studied psychological constructs: emotional reactivity, reward sensitivity, and punishment sensitivity. With regards to emotional reactivity, the amygdala has been, for example, linked to the processing of emotional stimuli [124]. The most studied amygdala reactivity-mediating gene is the *5-HTTLPR* of the *SCL6A4* gene [125, 126], and people carrying the *short allele* of *5-HTTLPR* are generally more vulnerable when exposed to negative stimuli and thus, under some conditions, maybe more likely to develop affective disorders [127, 128]. The results of our 5-HTTLPR study analyses were consistent with this mechanism, indicating that the short allele carries a vulnerability to substance use disorder in individuals that are exposed to severe negative environmental conditions.

Pertaining to the construct of reward sensitivity, dopamine regulates goal-directed behaviors through mesolimbic dopaminergic circuits, which appear to significantly contribute to levels of motivation for acquiring and consuming drugs [129]. Lowered reward sensitivity may also be a risk factor for thrill-seeking behaviors [129]. In impoverished environments with fewer available rewarding stimuli, direct and powerful rewards can be augmented, thereby resulting in somewhat predictable patterns of substance use and misuse [87].

In compliance with this theory, high-activity alleles of *MAOA*, which codes for an enzyme that is also involved in the degradation of dopamine, *DRD4* seven-repeat allele, *DRD2 A1* allele, and *DAT* 10-repeat allele are hypothesized as additional risk alleles, but results are inconsistent. Most of the included studies advocated *MAOA* low-activity allele as the risk allele. In DRD studies, the DRD4 seven-repeat and DRD2 A1 allele were indicated as risk alleles, which are consistent with a dopamine-linked theoretical model presented above. It is possible that the MAOA gene effect observed here contradicts any expected patterns of gene outcomes, but discrepancies might be accounted for by heterogeneity in population selections or the range of differential measurements applied.

For punishment sensitivity, serotonin and dopamine appear to coordinate their actions in this behavioral process. Low punishment sensitivity can function asa risk factor for various psychopathies or substance use behavior [130, 131]. Moreover, unpleasant family environments can increase insensitivity to punishment, which may result in being less concerned about behavior consequences and may manifest as in reward-driven behavior [87]. The *5-HTTLPR L-allele* and *MAOA* high-activity allele are indicated to be the risk allele for punishment insensitivity. For *5-HTTLPR*, emotional reactivity seems to be the dominant mechanism rather than punishment sensitivity because *S-allele* is more often supported as the risk allele for substance use. For *MAOA*, the results were mixed, and further investigations are needed in this field.

### General discussion

Among the 44 studies, 38 (86.36%) reported as least one significant G×E effect on substance abuse, and the proportion of significant G×E effect is similar to a previous review focusing on cigarette use only (i.e., 87.5%) [14]. However, the mixed results across studies and the high heterogeneity in study design and measurement make it difficult to draw definitive conclusions about the existence of specific G×E effects for these genes. In addition, some studies showed sex dimorphism [63], whereas others did not. Moreover, publication biases might exist that lend to challenges in concluding G×E effects. Overall, large methodological differences continue to provide obstacles when conducting meta-analyses or even when providing less complex cross-study comparisons.

### Limitations of the included studies

#### rGE (gene–environment correlation)

rGE, which refers to the phenomenon where individuals’ genotype also influences their exposure to the environment, is less commonly seen in the studies included in our review. Reporting rGE is important because the environmental exposure can be shaped by genetical risk, which may be a source of confounder [5]. Fifteen out of 44 studies accounted for rGE, which may be considered as a relatively low proportion. Future studies may benefit from considering whether it is important to take rGE into account rather carefully in an effort to improve validity measurements.

#### Power calculation

Calculating the sample size in studies is a critical issue that determines the validity and contribution of the study. For many of the studies included in this review, power calculations are oftentimes not reported. A little less than 25 percent of the current studies report to utilize power calculations, and it is possible that some remaining studies were therefore underpowered when reporting insufficient effect sizes. Underpowered sample sizes directly impact on the strength of hypothesis tests, and the use of insufficient sample sizes may increase the likelihood of type II errors, along with any associated economic losses and lack of scientific gains [132]. We recommend future research to consider the use of careful power calculations beforehand to achieve the maximal validity of the study.

#### Study design

The candidate gene method has been criticized for its immature view of the effect of a single gene on a complicated biological pathway. Therefore, at present, several researchers use CGS or GWAS to better understand the role of genes in the development of various complex behaviors. Thus, we also conducted a preliminary search of CGS and GWAS, and such studies are summarized in S3 Table. Among six included CGS studies, four reported a statistically significant interaction effect. Among 21 included GWAS, only nine reported a statistically significant result. A similar issue was also observed by Pasman et al. [5], that is, the polygenic risk methods cannot always generate consistent findings. In addition, GWAS will determine many genes with unknown pathway, thereby causing difficulties in interpreting results and understanding the functional involvement of GWAS contributions. Importantly, it does not appear that GWAS methods extend to the precision of theories concerning the hypothetical constructs of reward sensitivity, punishment sensitivity, or emotional reactivity. By contrast, the CGS method can overcome the aforementioned problems because it selects target genes based on some of the hypotheses and biological pathways, making it possible to interpret and fit into behavioral mechanisms. However, studies regarding the CGS method remain insufficient to draw any conclusion; thus, the selection of genes can only be based on a limited body of knowledge, which might not be accurate enough.

#### Heterogeneity

The heterogeneity of included studies limited this review from integrating the findings and generating more solid conclusions. The included studies varied in population selection, environmental exposure selection, environmental exposure measurement, outcome selection, and measurement, as well as in the way they test the G×E effect. These methodological heterogeneities make it hard to pool such studies and synthesize findings.

### Conclusion

Future studies that replicate previous studies using similar environmental exposure, outcomes, and measurements to facilitate future pooled analysis may be highly beneficial. Conducting power calculation can determine the ideal sample size before starting research to achieve maximal validity. Analyzing and reporting rGE are important as it can minimize the potential confounders. Moreover, adjusting for various covariates such as age, gender, and ethnicity should be encouraged in future analyses. Researchers may wish to prioritize studies using selective theory-based approaches and to stringently control for multiple comparisons when observing complex statistical analyses.

## Supporting information

S1 Checklist (DOCX)

S1 Table Search terms. (DOCX)

S2 Table The p values of studies. (DOCX)

S3 Table Polygenic methods. (DOCX)
