# Supplementary material for: Candidate gene-environment interactions in substance abuse: A systematic review
Source: PLoS One. 2023 Oct 31;18(10):e0287446. doi: 10.1371/journal.pone.0287446 (PMC10617739; doi:10.1371/journal.pone.0287446)
Supplement: S1 Table — (DOCX) [file pone.0287446.s002.docx]

**S1 Table. Search terms**

| Substance use | Gene-environment |
| --- | --- |
| Substance abuse | Genotype environment |
| Alcohol | GxE |
| Ethanol | gene interaction |
| Smok* | Gene moderat* |
| Cigarette | Gene inter* |
| Tobacco | Genotype interplay |
| Cannabis | Genotype moderat* |
| Marijuana |  |
| Marihuana |  |
| Cocaine |  |
| MDMA |  |
| LSD |  |
| Methamphetamine |  |
| Nicotine |  |
| Heroin |  |
| Dependen* |  |
| Addict* |  |

Between columns, AND was used for separation, between rows, OR was used for separation
