# Supplementary material for: Candidate gene-environment interactions in substance abuse: A systematic review
Source: PLoS One. 2023 Oct 31;18(10):e0287446. doi: 10.1371/journal.pone.0287446 (PMC10617739; doi:10.1371/journal.pone.0287446)
Supplement: S2 Table — (DOCX) [file pone.0287446.s003.docx]

**S2 Table. The p values of studies.**

| Authors | Year | Design | P value | Bias |
| --- | --- | --- | --- | --- |
| Bau[25] | 2000 | DRD2 Taq1 A1 X stress on physiological dependence  DRD2 Taq1 A1 X harm avoidance on physiological dependence | p=0.004  p=0.05 | confounding bias, selection bias |
| Madrid[17] | 2001 | DRD2 Taq1 A1 X total Stress score on alcoholism score  DRD2 Taq1 A1 X occupational/economical stress score on alcoholism score | p<0.05  p=0.003 | confounding bias, selection bias, recall bias |
| Nilsson[26] | 2005 | 5-HTTLPR X family relation on high intoxication frequency | p=0.05 | confounding bias, selection bias, analysis reporting bias |
| Lerer^#^[27] | 2006 | HTR6-C276T genotype X lifetime traumatic experience on nicotine dependence and smoking initiation | p=0.0001 | confounding bias, recall bias |
| Dick[30] | 2006 | 5-HTTLPR X stressful life events | p=NA | confounding bias, selection bias |
| Kaufman[28] | 2007 | 5-HTTLPR X Maltreatment for alcohol use | p=0.0001 | confounding bias, recall bias, selection bias |
| Covault^*^[29] | 2007 | 5-HTTLPR X stressful life events on drinking frequency year 1  5-HTTLPR X stressful life events on drinking frequency year 2  5-HTTLPR X stressful life events on heavy drinking frequency year 1  5-HTTLPR X stressful life events on heavy drinking frequency year 2  5-HTTLPR X stressful life events on drinking intentions year 1  5-HTTLPR X stressful life events on drinking intentions year 2  5-HTTLPR X stressful life events on proportion of drug use days year 1  5-HTTLPR X stressful life events on proportion of drug use days year 2 | p=0.031  p=0.029  p=0.011  p=0.016  p=0.025  p= .033  p=0.041  p=0.029 | confounding bias, recall bias, selection bias |
| Nilsson[15] | 2007 | MAOA X Merged psychosocial variable on alcohol related problem behavior  MAOA X Family relations on alcohol related problem behavior  MAOA X Maltreatment/abuse on alcohol related problem behavior | p=0.011  p=0.085  p=0.086 | confounding bias, selection bias, analysis reporting bias |
| Dick[13] | 2007 | GABRA2 X marital status | p=0.004 | confounding bias, selection bias |
| Segman^#^[31] | 2007 | DAT1 C-9 haplotype X lifetime traumatic experience on nicotine dependence | p=0.01 | confounding bias, selection bias, recall bias |
| Vanyukov[31] | 2007 | MAOA haplotype X parenting indices on substance use disorder | p=0.0320 | confounding bias, recall bias |
| Blomeyer[14] | 2008 | CRHR1 rs242938 X negative life events on Current Monthly Drinking  CRHR1 rs242938 X negative life events on Lifetime Heavy Drinking  CRHR1 rs242938 X negative life events on Average Amount of Alcohol Consumed/Month  CRHR1 rs242938 X negative life events on Maximum Amount of Alcohol Consumed/Occasion  CRHR1 rs1876831 X negative life events on Current Monthly Drinking  CRHR1 rs1876831 X negative life events on Lifetime Heavy Drinking  CRHR1 rs1876831 X negative life events on Average Amount of Alcohol Consumed/Month  CRHR1 rs1876831 X negative life events on Maximum Amount of Alcohol Consumed/Occasion | p=0.166  p=0.440  p=0.653  p=0.169  p=0.496  p=0.024  p=0.237  p=0.030 | confounding bias, selection bias |
| Gacek^*^[33] | 2008 | TPH1 X Negative life events on alcohol consumption and drinking frequency  TPH2 X Negative life events on alcohol consumption and drinking frequency  5-HTTLPR X Negative life events on alcohol consumption and drinking frequency | p=NA  p=NA  p=NA | confounding bias, recall bias, selection bias |
| Nilsson[34] | 2008 | MAOA X quality of family relations on AUDIT index  MAOA X maltreatment on AUDIT index:  MAOA X quality of family relations on Alcohol-related problem behaviour index  MAOA X maltreatment on Alcohol-related problem behaviour index | p=0.001  p=ns.  p=0.001  p=ns. | confounding bias, selection bias, analysis reporting bias |
| Ducci[35] | 2008 | MAOA haplotype B X Childhood sexual abuse on alcoholism  MAOB haplotype C X Childhood sexual abuse on alcoholism | p=0.03  p=0.14 | confounding bias, recall bias |
| Chen[36] | 2009 | CHRNA5 rs16969968 X parent monitoring on nicotine dependence  CHRNA3 rs3743078 x parent monitoring on nicotine dependence | p=0.034  p=0.80 | confounding bias, selection bias, analysis reporting bias |
| Du[37] | 2009 | OPRM1 X Marriage X Education on overall maxdrinks  OPRM1 X 5-HTTLPR X Marriage X Education on overall maxdrinks  OPRM1 X HTTLPR X DRD2 X Marriage X Education on overall maxdrinks  OPRM1 X Marriage X Education on maxdrinks>=36  OPRM1 X HTTLPR X DRD2 X Education on maxdrinks>=36  OPRM1 X Education on maxdrinks>=36  OPRM1 X HTTLPR X DRD2 X Marriage X Education on maxdrinks>=36  5-HTTLPR X Marriage X Education on maxdrinks in 23-35  OPRM1 X 5-HTTLPR X Marriage X Education on maxdrinks in 23-35  OPRM1 X HTTLPR X DRD2 X Marriage X Education on maxdrinks in 23-35  HTTLPR X DRD2 X Marriage on maxdrinks <=22  OPRM1 X 5-HTTLPR X DRD2 X Marriage on maxdrinks <=22  OPRM1 X HTTLPR X DRD2 X Marriage X Education on maxdrinks <=22 | p=0.1719  p=0.632  p=0.632  p=0.0547  p=0.0547  p=0.0107  p=0.0547  p=0.0547  p=0.632  p=0.377  p=0.623  p=0.9893  p=0.999 | confounding bias, recall bias |
| Laucht[38] | 2009 | 5-HTTLPR X Adversity on binge drinking days  5-HTTLPR X Life Events on binge drinking days  5-HTTLPR X Adversity on number of drinks  5-HTTLPR X Life Events on number of drinks | p=0.059  p=0.042  p=0.099  p=0.001 | confounding bias, recall bias |
| Schmid[39] | 2010 | CRHR1 rs1876831 X stressful life events on age at first drink  CRHR1 rs242938 X stressful life events on age at first drink  CRHR1 rs1876831 X stressful life events on No. of drinks  CRHR1 rs242938 X stressful life events on No. of drinks  CRHR1 rs1876831 X stressful life events on maximum number of drinks/occasion  CRHR1 rs242938 X stressful life events on maximum number of drinks/occasion  CRHR1 rs1876831 X stressful life events on drinking days  CRHR1 rs242938 X stressful life events on drinking days  CRHR1 rs1876831 X stressful life events on binge-drinking days  CRHR1 rs242938 X stressful life events on binge-drinking days | p=0.035  p=0.542  p=0.028  p<0.001  p=0.462  p=0.08  p=0.932  p=0.197  p=0.053  p=0.001 | confounding bias, recall bias |
| van der Zwaluw[40] | 2010 | DRD2 X rules from T1 to T2  DRD2 X rules from T2 to T3 | p<0.05  p<0.01 | confounding bias, recall bias |
| Nelson[41] | 2010 | CRHR1 H2 haplotype X Childhood sexual abuse on alcohol dependence risk  CRHR1 H2 haplotype X Childhood sexual abuse on alcohol use | p=0.023  p<0.017 | confounding bias, recall bias |
| Enoch[18] | 2010 | GABRA2 X Childhood trauma on cocaine use  GABRA2 X Childhood trauma on substance+ alcohol | p=0.039  p=0.076 | confounding bias, recall bias |
| Ducci[42] | 2011 | TTC12 rs10502172 X novelty seeking at 31 years old on smoking  CHRNA3 rs1051730 X novelty seeking at 31 years old on smoking  TTC12 rs10502172 X maternal smoking on smoking regularly at 14 years old  TTC12 rs10502172 X maternal smoking on smoking occasionally at 14 years old  TTC12 rs10502172 X maternal smoking on smoking heavily at 31 years old  TTC12 rs10502172 X maternal smoking on light-smoking at 31 years old  CHRNA3 rs1051730 X maternal smoking on smoking occasionally at 14 years old  CHRNA3 rs1051730 X maternal smoking on smoking regularly at 14 years old  CHRNA3 rs1051730 X maternal smoking on heavy smoking at 31 years old  CHRNA3 rs1051730 X maternal smoking on light smoking at 31 years old | p=0.06  p=0.78  p= 0.61  p=0.48  p=0.20  p=0.81  p=0.003  p=0.23  p=0.59  p=0.48 | confounding bias, recall bias |
| Kranzler[43] | 2011 | CRHR1 X Childhood adverse events on alcohol dependence in African American male  CRHR1 X Childhood adverse events on alcohol dependence in African American female  CRHR1 X Childhood adverse events on alcohol dependence in European American male  CRHR1 X Childhood adverse events on alcohol dependence in European American female | p=0.61  p=0.17  p=0.13  p=0.38 | confounding bias, recall bias |
| Fletcher[44] | 2012 | CHRNA6 GG subtype X per-pack tobacco tax rate on smoking  CHRNA6 GC subtype X per-pack tobacco tax rate on smoking  CHRNA6 CC subtype X per-pack tobacco tax rate on smoking | p=0.0148  p=0.41  p=0.37 | confounding bias, selection bias, recall bias |
| Xie[45] | 2012 | CHRNA5 rs16969968 X childhood adversity on alcohol dependence in men  CHRNA5 rs16969968 X childhood adversity alcohol dependence in women | p=0.0044  p=0.61 | confounding bias, recall bias |
| Vaske[46] | 2012 | 5-HTTLPR X neglect on marijuana use | p<0.001 | confounding bias, selection bias |
| Daw[47] | 2013 | 5-HTTLPR*S X school drinking on drinks consumed(full sample)  5-HTTLPR*S X school drinking on drinks frequency(full sample)  5-HTTLPR*S X school drinking in drinks consumed(sibling sample)  5-HTTLPR*S X school drinking in drinks frequency(sibling sample)  5-HTTLPR*S X school smoking in cigarettes smoked(full sample)  5-HTTLPR*S X school smoking in cigarettes smoked(sibling sample)  5-HTTLPR*S X school smoking in smoking frequency(full sample)  5-HTTLPR*S X school smoking in smoking frequency(sibling sample) | p<0.05  p=ns.  p<0.01  p<0.01  p<0.05  p<0.05  p<0.05  p=ns | confounding bias, selection bias |
| Perry[48] | 2013 | GABRA2 rs279871 AA X positive daily experiences on alcohol dependence in male  GABRA2 rs279871 AA X positive daily experiences on alcohol dependence in male | p<0.05  p=ns | confounding bias, recall bias |
| Miranda[49] | 2013 | OPRM1 X Deviant Peer Affiliation in alcohol use disorder  OPRM1 X Parental Monitoring in alcohol use disorder | p=0.001  p=0.004 | confounding bias, recall bias |
| Ray[50] | 2013 | CRHR1 Block1 H1 X Trauma on alcohol dependence  CRHR1 Block1 H3 X Trauma on alcohol dependence  CRHR1 Block2 H1 X Trauma on alcohol dependence  CRHR1 Block2 H2 X Trauma on alcohol dependence  CRHR1 Block2 H3 X Trauma on alcohol dependence  CRHR1 Block2 H5 X Trauma on alcohol dependence  CRHR1 Block2 H6 X Trauma on alcohol dependence  CRHR1 Block2 H6 X Trauma on alcohol dependence | p=0.029  p=0.15  p=0.026  p=0.63  p=0.43  p=0.078  p=0.48  p=0.042 | confounding bias, recall bias |
| Olsson[23] | 2013 | DRD4 X insecure attachment on cannabis use  DRD4 X insecure attachment on tobacco use  DRD4 X insecure attachment on alcohol use | p=0.006  p=0.240  p=0.080 | confounding bias, recall bias |
| Hiemstra[51] | 2014 | Study1:  DRD2 X maternal smoking on smoking  DRD4 X maternal smoking on smoking  DAT1 X maternal smoking on smoking  DRD2 X paternal smoking on smoking  DRD4 X paternal smoking on smoking  DAT1 X paternal smoking on smoking  DRD2 X sibling smoking on smoking  DRD4 X sibling smoking on smoking  DAT1 X sibling smoking on smoking  DRD2 X friends smoking on smoking  DRD4 X friends smoking on smoking  DAT1 X friend smoking on smoking  DRD2 X best friend smoking on smoking  DRD4 X best friend smoking on smoking  DAT1 X best friend smoking on smoking  Study2:  DRD2 X maternal smoking on smoking  DRD4 X maternal smoking on smoking  DAT1 X maternal smoking on smoking  DRD2 X paternal smoking on smoking  DRD4 X paternal smoking on smoking  DAT1 X paternal smoking on smoking  DRD2 X sibling smoking on smoking  DRD4 X sibling smoking on smoking  DAT1 X sibling smoking on smoking  DRD2 X friends smoking on smoking  DRD4 X friends smoking on smoking  DAT1 X friend smoking on smoking  DRD2 X best friend smoking on smoking  DRD4 X best friend smoking on smoking  DAT1 X best friend smoking on smoking | p=0.04  p=0.89  p=0.10  p=0.81  p=0.25  p=0.86  p=0.80  p=0.27  p=0.44  p=0.88  p=0.06  p=0.79  p=0.97  p=0.82  p=0.64  p=0.77  p=0.38  p=0.78  p=0.64  p=0.95  p=0.18  p=0.89  p=0.24  p=0.91  p=0.57  p=0.32  p=0.82  p=0.26  p=0.68  p=0.86 | selection bias |
| van der Zwaluw[52] | 2014 | OPRM1 X Rules in heavy vs light drinker  OPRM1 X Rules in heavy vs moderate drinker | p<0.001  p=0.055 | confounding bias, recall bias |
| Handley[9] | 2015 | FKBP5 X childhood maltreatment on marijuana use | p=0.04 | confounding bias, recall bias |
| Rovaris[53] | 2015 | NR3C2 rs5522 X physical neglect on cocaine use  NR3C2 rs5522 X emotional neglect on cocaine use  NR3C2 rs5522 X emotional abuse on cocaine use  NR3C2 rs5522 X sexual abuse on cocaine use  NR3C2 rs5522 X physical abuse on cocaine use  NR3C1 rs6198 X physical neglect on cocaine use | p=0.001  p= 0.004  p=0.521  p=0.438  p=0.728  p=0.198 | confounding bias, recall bias, analysis reporting bias |
| Windle[54] | 2016 | 5-HTTLPR X residential stability on substance use  5-HTTLPR X Neighborhood concentrated disadvantage on substance use  5-HTTLPR X Age X residential stability  5-HTTLPR X Age X Neighborhood concentrated disadvantage | 0.05<p<0.1  p<=0.05  p<=0.05  0.05<p<0.1 | confounding bias, analysis reporting bias |
| Bendre[55] | 2018 | MAOA X maltreatment on AUDIT  MAOA X maltreatment on AUDIT-C | p=0.015  p=0.052 | selection bias |
| Fite^$^[56] | 2019 | MAOA X maltreatment X sex | p= 0.00 | confounding bias, recall bias |
| Fite^$^[57] | 2019 | MAOA-L X any maltreatment type on polysubstance use in male  MAOA-L X emotional abuse on polysubstance use in male  MAOA-H X any maltreatment type on polysubstance use in male  MAOA-H X physical abuse on polysubstance use in male  MAOA-LL and MAOA-LH X any maltreatment type on number of substance used in female  MAOA-LL and MAOA-LH X physical abuse on number of substance used in female  MAOA-LL and MAOA-LH X emotional abuse on number of substance used in female  MAOA-HH X any maltreatment type on number of substance used in female  MAOA-HH X physical abuse on number of substance used in female  MAOA-HH X emotional abuse on number of substance used in female | p = 0.08  p = 0.03  p = 0.07  p = 0.03  p = 0.99  p = 0.18  p = 0.17  p = 0.04  p = 0.03  p = 0.04 | confounding bias, selection bias |
| Su[58] | 2019 | 5-HTTLPR X parenting on alcohol use frequency and quantity in males  5-HTTLPR X parenting on alcohol use frequency and quantity in females | p=0.008  p=0.766 | confounding bias, recall bias, analysis reporting bias |
| Navarro-Mateu[59] | 2019 | 5-HTTLPR X Maltreatments and family negativities on alcohol/drug abuse | p=ns. | confounding bias |
| Hendershot[60] | 2020 | OPRM1 X adherence on same-day craving  OPRM1 X adherence on drinks per day | p=0.06  p=0.007 | confounding bias, recall bias |
| Ossola[61] | 2021 | 5-HTTLPR X adverse child experience on alcohol use disorder  DRD2 Taq1A X adverse child experience on alcohol use disorder | p=0.959  p=0.531 | confounding bias, recall bias, selection bias |
