# Supplementary material for: Candidate gene-environment interactions in substance abuse: A systematic review
Source: PLoS One. 2023 Oct 31;18(10):e0287446. doi: 10.1371/journal.pone.0287446 (PMC10617739; doi:10.1371/journal.pone.0287446)
Supplement: S3 Table — (DOCX) [file pone.0287446.s004.docx]

**S3 Table. Polygenic methods.**

| Authors | Title | Year | Study design | Method Environmental  exposure | | Outcome | Interaction? |
| --- | --- | --- | --- | --- | --- | --- | --- |
| Guo[20] | Peer influence, genetic propensity, and binge drinking: a natural experiment and a replication. | 2015 | Longitudinal | Candidate gene score | Roommate binge drinking in college | Self-report binge drinking in college | Yes |
| Bountress[121] | Parent and peer influences on emerging adult substance use disorder: a genetically informed study. | 2016 | Longitudinal | Candidate gene score | Parental knowledge & peer substance use | Substance use disorder diagnosed by Diagnostic Interview Schedule | Yes |
| Stogner[122] | Genetic modification of the relationship between parental rejection and adolescent alcohol use. | 2016 | Correlational | Candidate gene score | Parental rejection | Self-report alcohol use initiation | Yes |
| Pasman[123] | Hypodopaminergic polygenic risk for polysubstance use. | 2017 | Longitudinal | Candidate gene score | Parental education level | Smoking determined by FTND, problematic alcohol use determined by either CAGE or RAPI, cannabis or other substance use based on self-report | Yes |
| Kim[124] | Interaction Effects between the Cumulative Genetic Score and Psychosocial Stressor on Self-Reported Drinking Urge and Implicit Attentional Bias for Alcohol: A Human Laboratory Study | 2019 | Correlational | Candidate gene score | Stress condition | Binge drinking determined by Alcohol Urge Questionnaire | No |
| Coley[125] | Environmental risks outweigh dopaminergic genetic risks for alcohol use and abuse from adolescence through early adulthood. | 2017 | Longitudinal | Candidate gene score | Parent and friend drinking & stressful life events | Alcohol use, intoxication and alcohol use disorder based on DSM-V | No |
| Vrieze[126] | The interplay of genes and adolescent development in substance use disorders: leveraging findings from GWAS meta-analyses to test developmental hypotheses about nicotine consumption. | 2012 | Correlational | GWAS | Age cohort | Self-report alcohol use and smoking heaviness | Yes |
| Meyers[127] | Interaction between polygenic risk for cigarette use and environmental exposures in the Detroit Neighborhood Health Study. | 2013 | Correlational | GWAS | Traumatic events & neighbourhood social cohesion | Self-report smoking heaviness | Yes |
| Salvatore[128] | Polygenic scores predict alcohol problems in an independent sample and show moderation by the environment. | 2014 | Correlational | GWAS | Parental knowledge & peer deviance | Alcohol problem determined by Child versionSSAGA | Yes |
| Musci[129] | Testing gene x environment moderation of tobacco and marijuana use trajectories in adolescence and young adulthood | 2015 | Longitudinal | GWAS | Friend's substance use & parent monitoring | Self-report frequency of substance use | Yes |
| Domingue[130] | Cohort effects in the genetic influence on smoking. | 2016 | Correlational | GWAS | Birth cohort | Self-report smoking initiation age | Yes |
| Schimitz[131] | The long-term consequences of Vietnam-era conscription and genotype on smoking behavior and health | 2016 | Case-control | GWAS | Veteran status & educational attachment | Self-report smoking initiation and heaviness | Yes |
| Li[132] | The impact of peer substance use and polygenic risk on trajectories of heavy episodic drinking across adolescence and emerging adulthood | 2017 | Longitudinal | GWAS | Close friend substance use | Self-report heavy episodic drinking | No |
| Treur[133] | Testing familial transmission of smoking with two different research designs. Nicotine Tobacco | 2017 | Correlational | GWAS | Childhood smoke exposure | Self-report smoking initiation and heaviness | No |
| Mies[134] | Polygenic risk for alcohol consumption and its association with alcohol-related phenotypes: do stress and life satisfaction moderate these relationships? | 2018 | Correlational | GWAS | Stress& life satisfaction | Self-report alcohol consumption and alcohol related problem determined by AUDIT | No |
| Polimanti[135] | Trauma exposure interacts with the genetic risk of bipolar disorder in alcohol misuse of US soldiers. | 2018 | Case-control | GWAS | Trauma exposure | Alcohol misuse and nicotine dependence determined by questionnaire | Yes |
| Marceau[136] | Interactions between Genetic, Prenatal, Cortisol, and Parenting Influences on Adolescent Substance Use and Frequency: A TRAILS Study | 2021 | Longitudinal | GWAS | Warm parenting | Self-report past-month alcohol, tobacco and cannabis use | No |
| Pasman[137] | Interplay between genetic risk and the parent environment in adolescence and substance use in young adulthood: A TRAILS study | 2021 | Longitudinal | GWAS | Warm parenting | Self-report past-month alcohol, tobacco and cannabis use | No |
| Zaso[138] | Effects of Polygenic Risk and Perceived Friends' Drinking and Disruptive Behavior on Development of Alcohol Use Across Adolescence | 2020 | Longitudinal | GWAS | Friend's drinking, friend's disruptive behavior | Self-reported drinking frequency | No |
| Salvatore[139] | Incorporating functional genomic information to enhance polygenic signal and identify variants involved in gene-by-environment interaction for young adult alcohol problems | 2018 | Correlational | GWAS | Relationship status | Self-report frequency of alcohol intoxication | No |
| Su[140] | Examining interactions between genetic risk for alcohol problems, peer deviance, and interpersonal traumatic events on trajectories of alcohol use disorder symptoms among African American college students | 2018 | Longitudinal | GWAS | Peer deviance, interpersonal traumatic events | Alcohol use disorder based on DSM-5 | No |
| Bares[141] | Exploring how Family and Neighborhood Stressors Influence Genetic Risk for Adolescent Conduct Problems and Alcohol Use | 2020 | Case-control | GWAS | Family and neighbourhood stressor | Self-report alcohol use, alcohol frequency | No |
| Smith[142] | Genes, Roommates, and Residence Halls: A Multidimensional Study of the Role of Peer Drinking on College Students' Alcohol Use | 2019 | Longitudinal | GWAS | Roommate alcohol use, residence hall alcohol use | Self-report alcohol use assessed by AUDIT-C | No |
| Su[143] | The associations between polygenic risk, sensation seeking, social support, and alcohol use in adulthood | 2021 | Longitudinal | GWAS | Social support | Self-report alcohol consumption | Yes |
| Meyers[144] | Psychosocial moderation of polygenic risk for cannabis involvement: the role of trauma exposure and frequency of religious service attendance | 2019 | Longitudinal | GWAS | Lifetime trauma exposure & frequency of religious service attendance | Self-report cannabis use and cannabis use disorder symptoms count according to DSM-V | Yes |
| Kandaswamy[145] | Predicting alcohol use from genome-wide polygenic scores, environmental factors, and their interactions in young adulthood | 2021 | Longitudinal | GWAS | Relationship with twin, CHAOS at home, relationship status, on benefits, education status, being a parent, socioeconomic status, peer victimisation, life events, Negative Childhood Experience, online bullying, conflict with the law, life satisfaction,healthy diet, BMI physical activity, athlete status, sleep quality, online media use, peer pressure, hassles, self-control, risk taking, aggression, purpose in life, volunteering, mood, general anxiety, antisocial behavior | Alcohol use assessed by AUDIT-C | No |
| Pasman[146] | Genetic Risk for Smoking: Disentangling Interplay Between Genes and Socioeconomic Status | 2021 | Correlational | GWAS | Socioeconomical status | Self-report tobacco use | Yes |
